# Inhibition of Human Amylin Aggregation: *In Silico* and *In Vitro* Studies

**DOI:** 10.1021/acsomega.5c02443

**Published:** 2025-10-31

**Authors:** Katarzyna Mizgalska, Ubaida Al.-Aani, Yaqoub Aljaidah, Dawid Panek, Ali Chaari, Marek Bajda

**Affiliations:** † Department of Physicochemical Drug Analysis, Faculty of Pharmacy, 49573 Jagiellonian University Medical College, Kraków, Medyczna 9, Cracow 30-688, Poland; ‡ 36579 Weill Cornell Medicine Qatar, Qatar Foundation, Education City, P.O. Box 24144, Doha , Qatar

## Abstract

Human islet amyloid polypeptide (hIAPP), also termed amylin, is an endocrine hormone that plays a key role in regulating blood glucose levels. Pathological conformational changes in amylin can lead to its aggregation into amyloid deposits, which are significant markers in the development of type 2 diabetes (T2D) and Alzheimer’s disease (AD). In this study, we explored 1-benzylamino-2-hydroxyalkyl derivatives as potential amylin aggregation inhibitors. These compounds have previously demonstrated activity against amyloid-β aggregation in AD. We conducted ThT and DLS assays to identify compounds 18 and 22 as the most active derivatives, inhibiting amylin aggregation with IC_50_ values of 3.04 and 2.71 μM, respectively. These compounds preserved small-sized oligomers, which exhibited reduced cytotoxicity compared to controls. The fluorescence quenching assay revealed that compounds 18 and 22 significantly quenched the intrinsic fluorescence of amylin without altering the emission spectra, indicating conformational changes without major modifications in the Tyr37 region. Binding and thermodynamic analyses indicated strong, spontaneous interactions dominated by hydrophobic forces. An *in silico* study compared the behavior of compound 18 (the most potent) and compound 9 (the least potent) in the ThT assay. Overall, compound 18 formed more interactions with amylin than compound 9 and remained attached to the peptide for a longer time during the simulation, more frequently stabilizing the α-helical fragments. This stabilization may help delay the transition into intermediate structures associated with amyloidogenic β-sheet formation. Our findings offer new insights into the aggregation process and may inform the design of more effective aggregation inhibitors.

## Graphical abstract

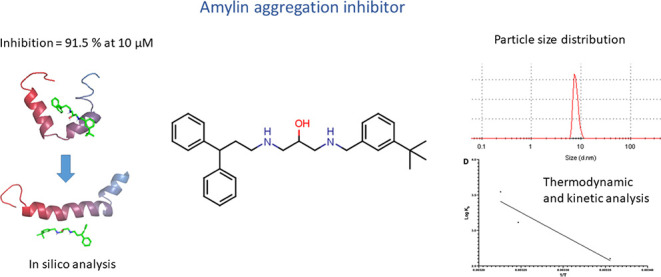

## 1 Introduction

Human islet amyloid polypeptide (hIAPP), henceforth referred to as amylin, is a 37-amino-acid peptide crucial in regulating blood glucose levels.^1^ Amylin participates in various physiological functions, such as inhibiting postprandial glucagon release, slowing gastric emptying, aiding glucose absorption, enhancing insulin action, and maintaining energy homeostasis as a satiating messenger.^2–5^ Despite its vital role in physiological processes, anomalous conformational alterations of amylin can contribute to pathological states. These changes, driven by yet unidentified factors, cause amylin to transition from a soluble to an insoluble form, leading to the accumulation of amyloid deposits, which are critical pathological markers in the pancreas for type 2 diabetes (T2D).^6,7^ Understanding factors associated with amylin conformational changes could lead to the development of therapeutic strategies to prevent the formation of insoluble amyloid deposits.^8–11^

Epidemiological research has shown that T2D is a risk factor for AD, with both conditions characterized by inflammation, abnormal metabolism, high oxidative stress, and amyloidogenic aggregates.^12–16^ Notably, the nature of the amyloid deposits varies between T2D and AD: in T2D, they are primarily composed of amylin found in the pancreas, whereas in AD, amyloid plaques in the brain consist mainly of amyloid-β (Aβ) peptide isoforms, specifically Aβ40 and Aβ42.^12,17^ Despite these compositional differences, many studies highlight similarities between amylin and Aβ peptides, such as a 25% identity in amino acid sequence and 50% similarity in conformation, which are particularly evident in the sequences that contribute to β-sheet formation—a structure abundant in amyloid deposits.^18^ Moreover, *in vitro* experiments have revealed that amylin can accelerate the aggregation of Aβ, suggesting a potential synergistic interaction between the two.^15,19,20^ Another key aspect linking T2D and AD is the disruption of proper insulin concentration. Under healthy conditions, insulin plays a crucial role in various cognitive processes within the cortex, hippocampus, and cerebellum. Furthermore, it directly regulates Aβ levels, thereby preventing its excessive accumulation in the brain.^21^ However, studies on insulin-like growth factors and their receptors have demonstrated abnormalities in insulin signaling among patients with AD.^22^ This connection between insulin dysfunction and cognitive decline has led researchers to refer to AD as “type 3 diabetes”, underscoring the shared pathologies of insulin signaling disturbances found in both dementia and diabetes.^22–25^

Early stages of T2D and AD are characterized primarily by oligomers. As these diseases progress, oligomers aggregate into larger, more structured amyloid fibrils.^16^ *In vitro* studies have demonstrated that amylin oligomers in the early stages of T2D are toxic, inducing apoptosis in pancreatic β-cells.^16,26–29^ Similarly, Aβ oligomers in AD have been shown to disrupt synaptic transmission, ultimately leading to neuron death.^12,17,30^ These findings highlight the urgent need for early-stage interventions targeting the oligomeric peptide forms to mitigate the most damaging impacts on cellular health effectively.

The assembly process of amylin is complicated, starting from simple monomers that aggregate to form oligomers and eventually mature into amylin fibrils. Nonpolar residues of amylin’s helical fragments constitute the hydrophobic surface, which drives the association of monomers. Influencing this process are π–π stacking interactions between aromatic amino acids, with residues Phe15, Phe23, and Tyr37 playing crucial roles in the self-assembly and molecular recognition of amylin monomers.^31^ Partially folded intermediates assemble into more structured oligomers, comprising the nuclei.^32^ The hydrophobic surface formed by residues 20–29 is critical for fibril development.^32^ As oligomerization progresses, interpeptide interactions lead to a network of intermolecular β-strands.^32,33^ The mature amylin fibrils are represented as the parallel arrangement of β-sheets, with hydrogen bonds oriented perpendicularly to both strands.^33^ Three aromatic residues, Phe15, Phe23, and Tyr37, are thought to be necessary for forming stable fibrils.^31,32^

The key method of amylin inhibitor investigation is the Thioflavin assay. Other investigations include the crystal violet oligomer indicator in amyloid aggregation,^34^ though these are less prevalent. These methods have been used in the identification of several inhibitors of amylin and amyloid.^34^ Many natural products are recognized as inhibitors of amylin aggregation. For instance, flavonoids such as chrysin and quercetin have demonstrated efficacy in inhibiting amylin aggregation in *in vitro* studies.^35^ Rosmarinic acid has also been identified as an amylin aggregation inhibitor, along with other catechol-containing compounds.^36^ Additionally, quercetin has been shown to protect pancreatic β-cells from damage induced by extracellular amyloid deposits.^37,38^ Similarly, other amyloid-inhibiting compounds have been identified through *in vitro* investigations,^39^ but the broader implementation of these inhibitors remains unclear. Furthermore, the stage at which these compounds intervene in inhibiting fibril formation and their mechanisms of action require further understanding. The critical reasons for investigating additional inhibitors lie in the limited specificity of the current small-molecule inhibitors of amylin, their limited efficacy, inability to reverse aggregation, and potential toxicity.^40,41^

Our research aims to assess the inhibitory effects and mechanisms of action of two groups of multifunctional compounds, known as multitarget-directed ligands (MTDLs), on amylin aggregation. These compounds, classified as 1-benzylamino-2-hydroxyalkyl derivatives, have previously demonstrated inhibitory activity against AD targets, such as AChE, BuChE, and BACE-1, as well as antiaggregation properties toward amyloid-β and tau. Specifically, such derivatives have shown promising dual aggregation inhibition of Aβ_42_ (inhibition range of 17.4 −80.0%) and tau (inhibition range of 38.0–73.6%) at a 10 μM screening concentration in a fluorescence ThS assay.^42,43^ Given that AD may be associated with brain insulin resistance in T2D and that Aβ and amylin share similarities in their amino acid sequences and utilize similar cytotoxic mechanisms in aggregation, we proposed investigating the effect of 1-benzylamino-2-hydroxyalkyl derivatives on amylin aggregation.

In this study, we performed *in vitro* and *in silico* studies on amylin aggregation for our MTDLs.^42,43^ We investigated the inhibition of amylin aggregation using two standard assays: Thioflavin T assay (ThT)^41,44–46^ and Dynamic Light Scattering assay (DLS).^44,45,47^ Furthermore, the particle size distribution was visualized by DLS assay. Compounds showing the highest and moderate activity in the ThT assay were subjected to IC_50_ evaluation. Additionally, we applied a fluorescence quenching assay to study tyrosine’s conformational change, which was thought to play a significant role in the aggregation process.^48,49^ Next, we analyzed the solvent accessibility of the peptide’s *C*-terminus during amylin aggregation using the Stern–Volmer equation^44,48–52^ and calculated thermodynamic parameters to assess the influence of hydrophobic interactions on binding.^49–52^ We then performed a cell viability assay to determine how the compounds affect amylin-induced cell toxicity. Next, we used the two-step autocatalytic aggregation model to evaluate which step of amylin aggregation the compounds inhibit the nucleation or the fibrillation stage.^53^ To further understand their molecular interactions, we compared the most *in vitro* active compound with the least active one using molecular docking and molecular dynamics simulations.^54,55^

## 2 Methods

### 2.1 Tested Multitarget-Directed Ligands (MTDLs)

MTDLs studied in this work were previously synthesized and tested *in vitro* against AD-related targets.^42,43^ The UPLC purity of these compounds was above 95%, and all were soluble in methanol and dimethyl sulfoxide solvents. The complete NMR characterization can be found in the published papers.^42,43^

### 2.2 Sample Preparation for *In Vitro* Experiments and Amylin Aggregation Protocol

A stock solution of amylin (1 mM) (purchased from Bachem USA), peptide sequence: KCNTATCATQRLANFLVHSSNNFGAILSSTNVGSNTY amide (with disulfide bond between Cys2 and Cys7), was prepared in 100% hexafluoroisopropyl alcohol (HFIP) (purchased from Sigma-Aldrich) and stored at −20 °C.^41,44^ Aliquots of amylin peptide were filtered through a 0.22 μm filter from the stock solution and freeze-dried. For kinetic experiments, 15 μM of amylin was prepared in 50 mM Tris-HCl buffer (pH 7.4), as investigated in previous studies by Chaari et al.,^41,44^ Samples of amylin were prepared in the presence or absence of inhibitors. Each compound was dissolved in DMSO and further diluted with the buffer used to solubilize the amylin. The final mixture contained 15 μM amylin, 10 μM of the test compound, 2% DMSO or 15 μM amylin, and 2% DMSO without the compound (negative control). Also 15 μM amylin, 10 μM of oleuropein aglycone and 2% DMSO was used as a positive control.^41^ The prepared solutions were incubated under physiological conditions (pH 7.4 and 37 °C) for amylin aggregation. Samples were incubated for 36 h to test for amylin aggregation, and measurements were conducted at different time points.^41^

### 2.3 Thioflavin T (ThT) Fluorescence Assay

Amylin aggregation was monitored by characteristic changes in Thioflavin T (ThT) fluorescence intensity. A ThT stock solution (15 μM) was prepared in deionized water at pH 7.4. The amylin samples were mixed with the ThT solution and allowed to incubate. ThT fluorescence was measured using the PerkinElmer model LS 55 fluorescence spectrophotometer (PerkinElmer, USA), with an excitation wavelength of 440 nm and an emission wavelength of 480 nm.^41,56–58^ The emission and excitation slits were set to 5 nm, and a 1 cm cuvette was used for all experiments. Nonspecific background fluorescence was subtracted from the samples using appropriate blanks in the absence of proteins. Each experiment was performed in triplicate.^41^

### 2.4 Dynamic Light Scattering (DLS) Assay

DLS (Zetasizer, Malvern) was used at a wavelength of 633 nm to measure the average diffusion coefficient distribution of amylin particles and the aggregation percentage. Samples were diluted 10-fold, and 50 μL was placed into a 96-well plate. The total light scattering intensity at a 90° angle was collected using a 10 s averaging acquisition time. Particle translational diffusion coefficients were calculated from autocorrelated light intensity data (usually 30–40 points) and converted to the hydrodynamic radius (*R*_h_) using the Stokes–Einstein equation. A distribution plot of intensity versus *R*_h_ was calculated using the Sedfit 9.3 analysis software, and intensity-weighted mean *R*_h_ values were obtained from each peak.^44^

### 2.5 Anilinonaphtalene-8-sulfonic Acid (ANS) Fluorescence Assay

Amylin hydrophobicity was monitored by characteristic changes in ANS fluorescence intensity during the aggregation process.^44,59^ Samples were diluted 50-fold with ANS solution (15 μM), and the mixture was incubated for 5 min at room temperature. ANS fluorescence was measured using a PerkinElmer model LS 55, with an excitation wavelength of 380 nm and an emission wavelength of 400 nm. The emission and excitation slits were set to 5 and 10 nm, respectively, and a 1 cm cuvette was used for all experiments. Nonspecific background fluorescence was subtracted from the samples by using appropriate blanks in the absence of proteins. Each experiment was performed in triplicate.^41^

### 2.6 Intrinsic Fluorescence Assay

The fluorescence emission scans (wavelengths 290 to 500 nm) of 10-fold diluted amylin samples were acquired with a PerkinElmer model LS 55. The excitation wavelength was set at 275 nm to observe the fluorescence of the tyrosine residue exclusively. The integrated spectrum from 290 to 450 nm was used to obtain the relative fluorescence values of Tyr. Fluorescence measurements were performed in 1 cm light-path quartz cuvettes, with both excitation and emission bandwidths set to 5 nm. Sample fluorescence was determined by subtracting the fluorescence of the buffer. Each experiment was performed in duplicate.^41,44^

### 2.7 Analysis of Binding Mechanism by Fluorescence Quenching of Amylin by Two Most Active Compounds

Quenching titrations with acrylamide were carried out by adding varying amounts of a quencher stock solution (5 M) to the protein solution (∼15 μM diluted 10-fold).^44,60^ The excitation wavelength was set at 275 nm, and the fluorescence emission spectra were scanned from 275 to 500 nm using a PerkinElmer model LS 55. The emission and excitation slits were set to 5 and 10 nm, respectively, and a 1 cm cuvette was used for all experiments. According to the Stern–Volmer equation, the integration area between 290 and 450 nm was used for data analysis.^44,61^ Before analyzing the quenching data, the fluorescence emission spectra were corrected for volume changes, scattering effects, and the inner filter effect due to acrylamide absorption.^44,61^ Each experiment was performed in duplicate.^44^ The quenching mechanism analysis by the Stern–Volmer equation was calculated according to eq 1.^62^ *F*_0_ is the fluorescence intensity without the quencher, and *F* is the intensity with a quencher. *K*_sv_ is the Stern–Volmer quenching constant, while *Q* is the quencher concentration. *K*_q_ is the biomolecular quenching constant, and τ_0_ is the average lifetime of the fluorophore.

1 $$\frac{F_{0}}{F}=1+K_{sv}\left[Q\right]=1+K_{q}\tau_{0}\left[Q\right]$$

Equation 2 was used to approximate the binding sites (*n*) and the binding constant (*K*_b_) of amylin with the two most active compounds. The parameter *n* obtained from the fit is treated as an apparent slope/exponent (heterogeneous populations and partial fluorophore accessibility) and is not interpreted as the literal number of binding sites.

2 $$\log\frac{\left(F_{0}-F\right)}{F}=\log K_{b}+n\log[Q]$$

The Van’t Hoff equation ([Disp-formula eq3]) was used to calculate the various thermodynamic interaction parameters.^62^ Equation 4 was implemented to calculate the free energy change.

3 $$\ln K_{b}=-\frac{\Delta H^{{}^{\circ}}}{RT}+\frac{\Delta S^{{}^{\circ}}}{R}$$

4 $$\Delta G^{{}^{\circ}}=\Delta H^{{}^{\circ}}-T\Delta S^{{}^{\circ}}=-RT\ln K_{b}$$

### 2.8 Kinetic Analysis

Kinetic analysis was performed according to the model of autocatalytic aggregation.^53^ According to Sabaté et al., aggregation is a two-step process, comprising nucleation (described by the *k*_n_ constant) and fibrillation phases (represented by the *k*_e_ constant). Two constants (*k*_n_ and *k*_e_) are calculated using the eq 5:

5 $$f=\frac{\rho \left\{e^{[\left(1+\rho \right)kt]}-1\right\}}{\left\{1+\rho e^{[\left(1+\rho \right)kt]}\right\}}$$

where *k* = *k*_e_ *a* (where *a* represents the initial concentration of the aggregating peptide), *f* stands for a fraction of the fibrillar form in the system, and ρ denotes the dimensionless ratio of *k*_n_ to *k*. The parameters *k* and ρ were calculated first, then *k*_n_ and *k*_e_.^53^

Data were normalized as a fraction of the highest fluorescence from the three trials and fitted into the model of autocatalytic aggregation. The same procedure was repeated for the remaining trials. The second calculation aimed to find the time parameters of the aggregation process: *t*_0_ (time point when the aggregation starts), *t*_1/2_ (aggregation halftime), and *t*_1_ (aggregation endpoint). *t*_0_, *t*_1/2_, and *t*_1_ were calculated as 10%, 50%, and 90% of the highest fluorescence measurements from the three trials, respectively. All calculations were performed using GraphPad Prism 9.5.1, Statistica 13.3.721.1, and Microsoft Excel 2304.

### 2.9 Cell Culture and Cytotoxicity Assay

In this study, we utilized Pancreatic INS-1E cells (Department of Cell Physiology and Metabolism, University Medical Center, Switzerland) due to their close physiological similarity to native β-cells,^63^ making them highly relevant to our experimental objectives. Pancreatic INS-1E cells were grown in RPMI 1640 medium, supplemented with 2 mM glutamine, 5% fetal calf serum, 10 mM HEPES (pH 7.4), 1 mM sodium pyruvate, 50 μM 2-mercaptoethanol, 100 units/mL penicillin, and 0.1 mg/mL streptomycin. The culturing environment was maintained at 37 °C, 5% CO_2_, pH 7.4.^44^

The toxicity of human amylin aggregates in the presence or absence of different compounds in the presence of 2% DMSO was assessed by the 3-(4,5-dimethylthiazol-2-yl)-2,5-diphenyltetrazolium bromide (MTT) reduction assay.^64^ The cells were cultured and then seeded into 96-well plates and grown for 24 h prior to exposure to different intermediates of amylin or a mixture of amylin with the MTDLs. The final amylin concentration within the culture medium was 5 μM. Cell viability was expressed as the percentage of MTT reduction, using cells treated with the same buffer volume as a reference (100% MTT reduction). The values (averages ± S.D.) were obtained from three independent experiments.^41^

### 2.10 *In Silico* Molecular Docking and Molecular Dynamics Simulations

The structure of amylin (PDB ID: 2L86 ^65^) was downloaded from the Protein Data Bank (PDB),^66^ as a Nuclear Magnetic Resonance (NMR) structure containing 20 possible conformations of the peptide, from which the first one was chosen for analysis. This structure includes all of the characteristic features of amylin used in *in vitro* studies. The peptide was prepared using the default preprocess options in the Protein Preparation Wizard^67^ module from the Schrödinger Suite 2020-3. The pH was adjusted to the physiological 7.4 ± 0.2 by Epik.^67^ Hydrogen bonds were optimized with the default options, and restrained minimization in the OPLS3e force field was applied.^67^ Amylin molecular systems were built through protein–protein docking performed using the pyDockWEB web server.^68^ The best-scored peptide complexes were selected for further study. The models of amylin fibrils were based on the structure downloaded from the PDB database under PDB ID: 7M65.^69^ This structure represents a cryogenic electron microscopy (Cryo-EM) structure of mature amylin fibrils extracted postmortem at a resolution of 4.10 Å. Five chains containing amylin’s core residues 6–37 were selected for the model. This structure was used in protein–protein docking to create the molecular system of five amylin β-strands associated with one α-helical monomer. The ligands for molecular docking were prepared using the LigPrep^70^ module of the Schrödinger Suite. Protonation states were assigned according to physiological pH 7.4 ± 0.2. All possible stereoisomers were generated for compounds with stereogenic centers. The protein grid box was centered on residues 20–30 for the systems of amylin monomers and included the whole structure for systems based on five amylin β-strands. Five poses were generated for each ligand. Molecular docking was performed using the Glide^71^ module of the Schrödinger Suite 2020-3 using the SP rigid docking protocol. The docking results were analyzed according to the Glide Score. The best-scored poses of the two chosen compounds in the S absolute stereoisomer were subjected to molecular dynamics simulations. Molecular dynamics simulations were performed solely on amylin systems and in combination with tested compounds. First, the simulation system was built using the System Builder^72^ module of the Schrödinger Suite. The TIP3P model was selected to represent water molecules, with the box shape set to orthorhombic with the default dimensions. The force field was set to OPLS3e, and the net charge of the structure was neutralized by adding a calculated number of Cl^–^ ions. Finally, sodium chloride was added to achieve a physiological concentration of 0.15 M. Desmond’s Molecular Dynamics^72^ module was implemented to run the simulation. The simulation time was 200 ns with a time step of 2 fs. The system was equilibrated according to the NPT ensemble class at a temperature of 25 °C and a pressure of 1 atm. The default relaxation protocol was implemented, and the seed for starting the simulation was set to random. Every system was simulated for 200 ns, except the three-monomer amylin system, which was subjected to a 500 ns simulation run. All simulations were performed in triplicate. The results were analyzed using Schrödinger Suite 2020-3 and VMD 1.9.3.

## 3 Results

### 3.1 Aggregation Inhibition Assays

To identify effective inhibitory activity, we evaluated two series of MTDLs, classified into series A and B, based on their slightly different scaffolds (Table 1). In series A, the nitrogen atom from the hydroxyalkylamine group is incorporated into a piperazine ring, whereas in series B, this nitrogen atom is part of an alkylamine fragment. Additionally, the R_1_ and R_2_ substituents are positioned at opposite ends of the molecule. The details behind the compounds’ design are described in previously published sources.^42,43^ The compounds were studied as racemates.

**Table 1:**
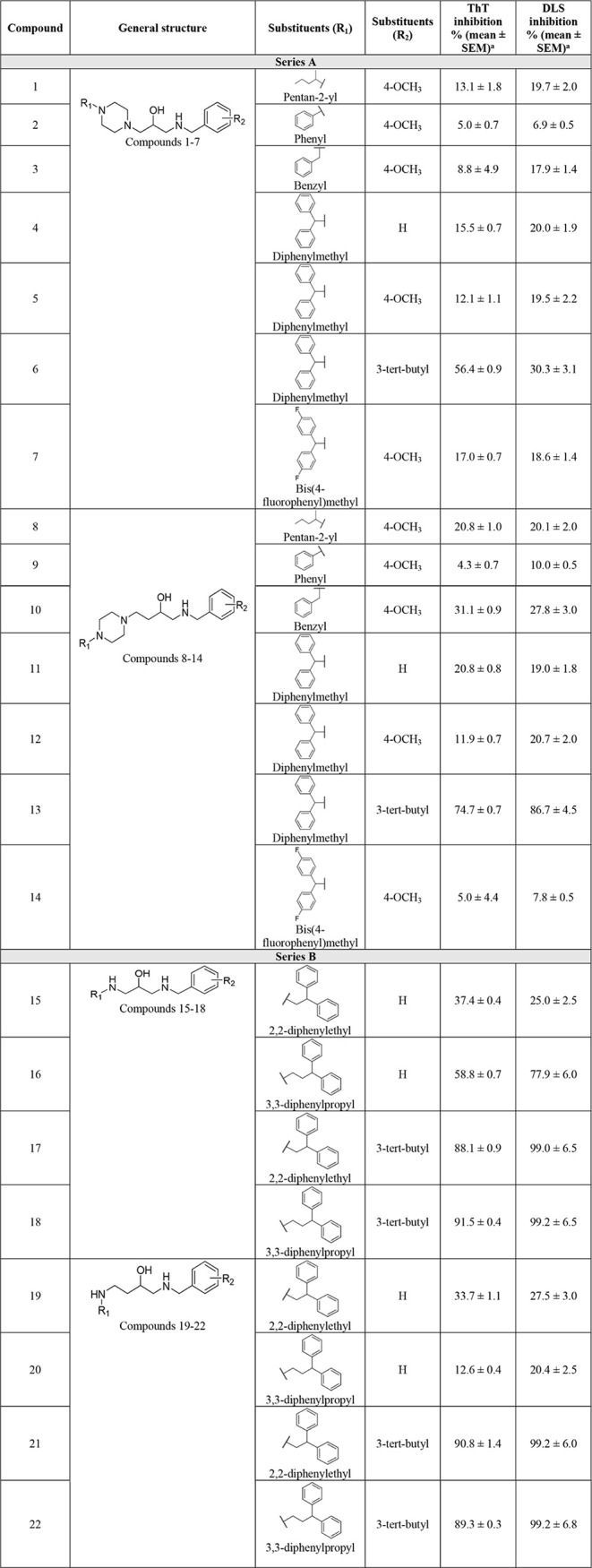
Inhibitory Activity in Aggregation Assays^a^

a Screening concentration of the compounds: 10 μM, amylin concentration: 15 μM, time of incubation: 36 h, number of trials: 3. Solubility data: all compounds were soluble in methanol and dimethyl sulfoxide solvents.

First, we assessed the effectiveness of our selected compounds in inhibiting amylin aggregation using both the ThT assay and the DLS techniques. The ThT assay revealed inhibition ranging from 4.3% to 91.5%, and the DLS assay showed inhibition between 6.9% and 99.2%. Notably, 7 out of 22 compounds inhibited more than 50% of amylin aggregation in both ThT and DLS assays, with compounds 17, 18, 21, and 22 exhibiting the highest inhibitory activity.

In the initial phase of our investigation, we focused on two series of compounds to identify the most effective combinations of R_1_ and R_2_ substituents and carbon chain lengths for inhibiting amylin aggregation. We started our analysis with series A, encompassing two groups of compounds sharing a similar basic structure but differing in the number of carbon atoms linking the hydroxyl group to the nitrogen atom in the piperazine structure: one group featuring two carbon atoms (compounds 1 to 7) and the other with three (compounds 8 to 14). When considering compounds 1 to 7 paired with a 4-OCH_3_ substituent as R_2_, we observed that compounds with smaller substituents, such as pentan-2-yl (compound 1), phenyl (compound 2), or benzyl (compound 3), exhibit lower inhibitory activity compared to larger ones, such as bis­(4-fluorophenyl)­methyl (compound 7). Hence, compound 6, featuring a diphenylmethyl substituent at the R_1_ position and a 3-*tert*-butyl substituent at the R_2_ position, demonstrated the highest inhibition activity (ThT: 56.4 ± 0.9, DLS: 30.3) among compounds 1 to 7. Notably, the presence of a third carbon atom linking the hydroxyl group to the nitrogen atom of the piperazine structure (compounds 8 to 14) further contributed to improving the inhibition activity of the compounds (compare compounds 1 with 8, 3 with 10, 4 with 11, and 6 with 13). The highest inhibition activity was observed in compounds 6 and 13, sharing identical R_1_ and R_2_ substituents (diphenylmethyl substituent and 3-*tert*-butyl, respectively), with compound 13 having one additional carbon atom compared to compound 6. Among these two, compound 13 exhibited the highest inhibition activity (ThT: 74.7% ± 0.7, DLS: 86.7%). The results are consistent with the Aβ aggregation studies conducted, where compounds 6 and 13 were the most active among those with a piperazine scaffold, showing inhibition rates of 77.2% ± 2.1 and 81.4% ± 7.9 in the ThT assay.^42^ Overall, we found that in series A, compounds with a 3-*tert*-butyl substituent at the R_2_ position consistently demonstrated the highest inhibitory activity, while those with 4-OCH_3_ at the R_2_ position were the least active. Therefore, the 3-*tert*-butyl substituent was carried over to series B, with 4-methoxy removed for further analysis.

Next, we shifted our focus to molecules in series B, which also consisted of two groups of compounds with a similar basic structure but varied in the number of carbon atoms linking the hydroxyl group with the basic nitrogen atom: one group containing two carbon atoms (compounds 15 to 18) and the other containing three (compounds 19 to 22). Series B contained the most active compounds. Since diphenylmethyl emerged as the most active R_1_ substituent in series A, it suggested the desired distance between the basic nitrogen atom and the aromatic substituent. To account for series B compounds no longer featuring a piperazine ring in the structure, the optimal length between the basic nitrogen and the aromatic substituent was achieved by introducing R_1_ substituents with longer linkers, such as 2,2-diphenylethyl and 3,3-diphenylpropyl.

Our analysis of compounds 15 to 18 revealed a trend of slightly lower activity for compounds with a 2,2-diphenylethyl as R_1_ substituent compared to those with a 3,3-diphenylpropyl as R_1_ substituent (compare compounds 15 with compounds 16 and 17 with 18). Interestingly, the effect for compounds 19–22 was the opposite, where compounds with a 2,2-diphenylethyl as the R_1_ substituent showed higher activity than those with a 3,3-diphenylpropyl as the R_1_ substituent. One possible explanation could be attributed to the length of the linker. The shorter linker, caused by the lower number of carbons, likely favors the 3,3-diphenylpropyl R_1_ substituent (compounds 16 and 18), whereas the longer linker prefers 2,2-diphenylethyl, maintaining the optimal distance between the terminating aromatic rings and the hydroxyl group. For the R_2_ substituents, we focused on two groups: 3-*tert*-butyl and hydrogen (H). We found that all compounds featuring 3-*tert*-butyl at the R_2_ position exhibited increased activity compared to those with H (compare 15 with 17, 16 with 18, 19 with 21, and 20 with 22), a trend also observed for series A compounds. Overall, we observed that compounds in series B showed higher inhibitory activity than series A, with compound 18 exhibiting the highest inhibition activity (ThT: 91.5% ± 0.4, DLS: 99.2%). Similarly to series A, series B also showed results comparable to the ThT Aβ aggregation study,^42^ where the most active compounds 17, 18, 21, and 22, achieved inhibition rates of 88.7% ± 5.5, 88.2% ± 5.9, 87.6% ± 3.1, and 84.9% ± 0.8, respectively. This suggests that these compounds exhibit similar inhibitory effects on both aggregating peptides Aβ and amylin, demonstrating their broad-spectrum activity.

Examining the chemical properties influencing the binding affinities of the compounds is crucial for understanding the molecular interactions that determine their efficacy in inhibiting peptide aggregation. Peptides that aggregate typically exhibit predominantly hydrophobic characteristics and contain only a few ionizable centers.^73,74^ Notably, series B compounds possess two ionization centers, while those in series A have three. This difference may account for the superior efficacy observed with series B compounds. Computationally calculated p*K*_a_ values for all the compounds can be found in the Supporting Information. Regarding hydrophobicity, the more active compounds are significantly more lipophilic, characterized by more aromatic rings and a *tert*-butyl substituent.^75^ These hydrophobic properties and fewer ionization centers in series B compounds contribute to a higher binding affinity, facilitating more effective interactions with the aggregating peptides. Overall, these insights underscore the importance of tailored molecular design in developing more potent inhibitors of peptide aggregation.^47^

Next, we calculated the IC_50_ values of compounds that inhibited amylin aggregation by 50% or more in the Thioflavin T assay and compared them to the positive control, oleuropein aglycone (Table 2). From series A, we selected the two compounds with the highest aggregation inhibition percentage values (compounds 6 and 13). From series B, we selected the compounds with inhibition percentage values >88% (compounds 17, 18, 21, and 22) and one compound with a moderately high inhibition percentage (compound 16). We found that compounds 18 and 22 had the lowest IC_50_ values, at 3.04 μM and 2.71 μM, respectively, compared to oleuropein aglycone, which has an IC_50_ of 1 μM. This result indicates that compound 22 is the most efficient in inhibiting amylin aggregation, followed by compound 18. In the Aβ aggregation study, IC_50_ values were calculated for compounds 18 and 22, based on the ThT assay results. For compound 18, a similar IC_50_ value was observed (IC_50_ = 3.09 μM). However, for compound 22, the IC_50_ was approximately 1.5 μM lower (IC_50_ = 1.22 μM), indicating a stronger inhibitory effect.^42^

**Table 2:** IC_50_ Values of the Selected Compounds

Compound	Oleuropein aglycone	6	13	16	17	18	21	22
**IC**_**50**_ **[μM]**	1	4.11	3.75	3.10	3.65	3.04	3.53	2.71
**ThT inhibition % (mean ± SEM)**	82.3 ± 2.0	56.4 ± 0.9	74.7 ± 0.7	58.8 ± 0.7	88.1 ± 0.9	91.5 ± 0.4	90.8 ± 1.4	89.3 ± 0.3

Monitoring protein aggregation through dynamic light scattering is well-established. Indeed, alterations in scattering intensity directly correlate with the increase in light intensity scattered by the soluble protein throughout the aggregation period.^76,77^ To study the inhibitory effects of the compounds further, we tested the particle size distribution using the DLS method, which is useful in detecting aggregate formation and size. We initially measured the particle size distribution of amylin to establish a baseline for its aggregation in the absence of any inhibitory compounds. This served as the control, where peaks were identified without the presence of inhibitors and then compared with peaks where inhibitors were present. At the start of the incubation, we observed an average particle size of 2 nm, corresponding to the native shape of the peptide (Figure 1A). Following 36 h of incubation, we recorded an average particle size of approximately 1000 nm (Figure 1B), indicating complete aggregation of amylin. Subsequently, we combined amylin with compounds exhibiting either low or high inhibition activity from previous experiments, and we compared it to oleuropein aglycone that demonstrated its ability to inhibit amylin aggregation as well as other amyloidogenic proteins.^41,78,79^ Compounds with low inhibition activity (compound 9) demonstrated an average particle size similar to that of the control (Figure 1C, compared to Figure 1B), validating their lack of effectiveness as potential aggregation inhibitors. Conversely, compounds with high inhibition activity (compounds 16, 17, 18, 21, and 22) exhibited an average particle size of 10 nm (Figure 1D) compared with the oleuropein aglycone (Figure 1E). These results indicated that the above compounds were able to interfere efficiently with the aggregation process, resulting in the formation of smaller-sized particles. These outcomes align with previous reports indicating that MTDLs can potentially hinder amyloid aggregation, maintaining the smaller size of intermediates and preventing the formation of large aggregates.

**Figure 1:**
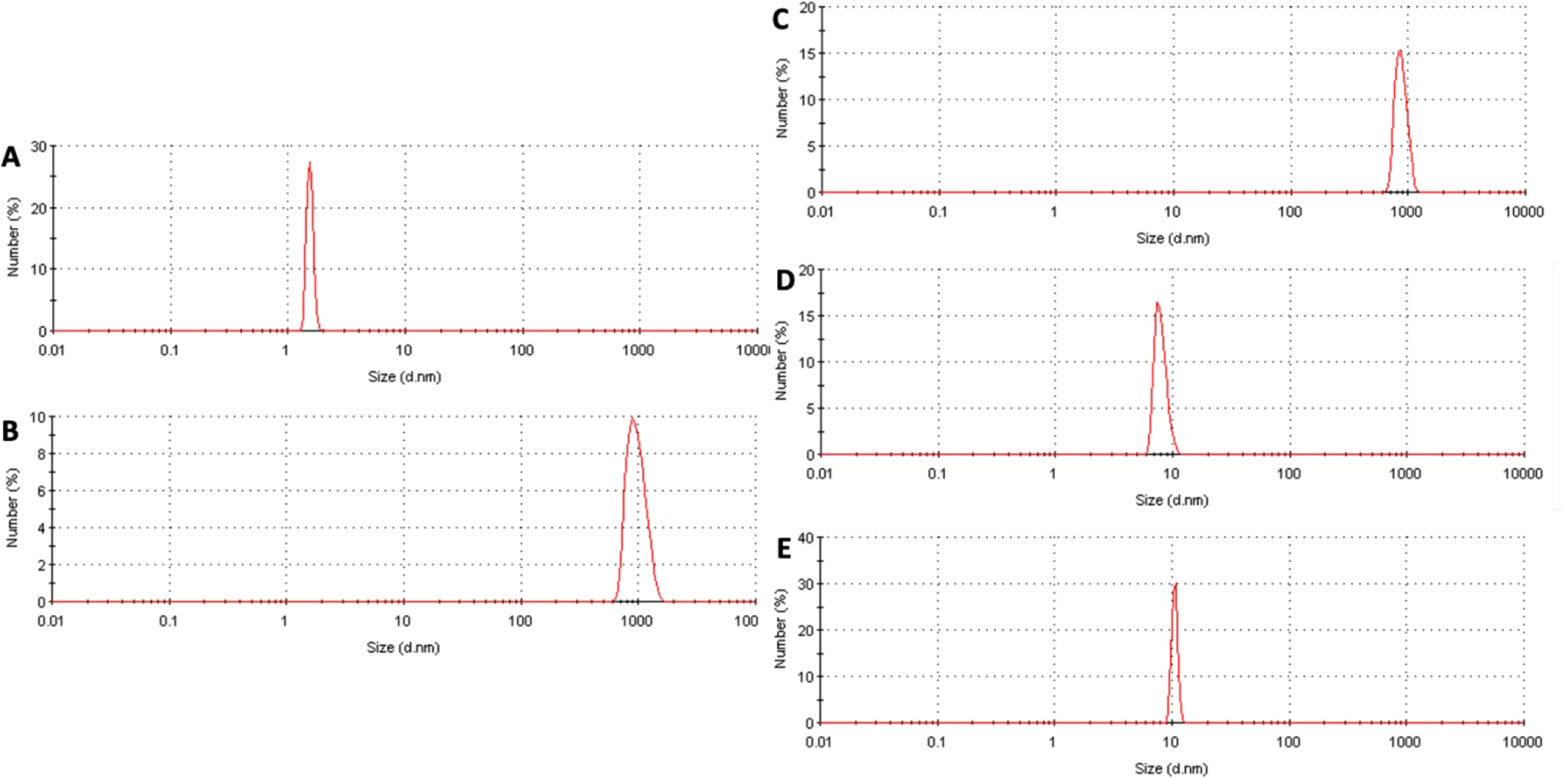
Particle size distribution for amylin determined by DLS: A) amylin (0 h), B) amylin (36 h), C) amylin + compound 9 (36 h), D) amylin + compound 18 (36 h), E) amylin + oleuropein aglycone (36 h).

### 3.2 Kinetics of Amylin Aggregation

Next, we conducted a kinetic assay to elucidate the mechanisms of action of the compounds with high inhibition efficacy. Specifically, we investigated the kinetic constants to identify the specific step of amylin aggregation that the tested compounds inhibit, whether it occurs during nucleation or elongation.^53,80^ We monitored the amylin aggregation kinetics in the absence or presence of the reference inhibitor, oleuropein aglycone, and compounds 17, 18, 21, and 22, using ANS and ThT assays. Compounds 6, 13, and 16 were also analyzed in the ThT assay. The kinetic parameters were calculated using the two-step autocatalytic aggregation model for ThT assay results (Table 3).

**Table 3:** Kinetic Parameters for Amylin Aggregation

Autocatalytic aggregation model^53^									
	Compounds								
Parameter (mean ± S.D.)^a^	Amylin	Oleuropein aglycone	6	13	16	17	18	21	22
**ρ** ^a^	0.0068 ± 0.0005	0.0093 ± 0.0002	0.0159±0.0044	0.0038 ± 0.0002	0.0042 ± 0.0003	0.0040 ± 0.0028	0.0186 ± 0.0051	0.0145 ± 0.0044	0.0624 ± 0.0126
*k* [h^–1^]^a^	0.2900 ± 0.0022	0.2366 ± 0.0075	0.2488 ± 0.0157	0.2729 ± 0.0025	0.2996 ± 0.0048	0.2687 ± 0.0374	0.2087 ± 0.0084	0.2273 ± 0.0221	0.2326 ± 0.0366
***k*_n_** [h^–1^]^a^	0.0020 ± 0.0002	0.0022 ± 0.0005	0.0039 ± 0.0009	0.0011 ± 0.0001	0.0012 ± 0.0001	0.0010 ± 0.0006	0.0039 ± 0.0009	0.0032 ± 0.0007	0.0142 ± 0.0006
***k***_e_ [M^–1^h^–1^]^a^	19331 ± 148	15773 ± 650	16585 ± 1051	18195 ± 165	19973 ± 320	17913 ± 2489	13913 ± 560	15155 ± 1477	15505 ± 2437
***t***_0_ [h]^a^	9.82 ± 0.21	10.76 ± 0.25	8.36 ± 0.56	12.49 ± 0.19	11.03 ± 0.22	13.13 ± 1.09	9.31 ± 0.72	9.54 ± 0.29	4.36 ± 0.16
***t***_**1**_ [h]^a^	24.67 ± 0.21	28.83 ± 0.30	25.27 ± 0.39	28.41 ± 0.23	25.52 ± 0.37	29.48 ± 1.02	29.29 ± 0.36	28.19 ± 1.42	20.69 ± 2.27
***t*** **1/2** [h]^a^	17.17 ± 0.21	19.67 ± 0.31	16.61 ± 0.27	20.41 ± 0.20	18.22 ± 0.28	21.25 ± 0.20	19.02 ± 0.57	18.66 ± 0.56	11.87 ± 1.02

a An average of at least three independent measurements.

The nucleation constant (*k*_n_) differed slightly among the compounds. The average *k*_n_ value for amylin equaled 0.002 h^–1^. The most active compounds, 18, 21, and 22, were characterized by similar nucleation constant values of 0.004, 0.003, and 0.014 h^–1^, respectively. A comparison of the elongation constant (*k*_e_) values for active compounds and the control revealed more significant differences. The average *k*_e_ for amylin was equal to 19331 M^–1^ h^–1^. The moderately active compound 6 had a *k*_e_ equal to 16585 M^–1^ h^–1^. In contrast, the most active compounds, 18, 21, 22, and the reference inhibitor oleuropein aglycone, were characterized by 13913, 15155, 15505, and 15773 M^–1^ h^–1^ values. The results suggest that the active compounds most likely inhibit amylin aggregation at the fibrillation stage. Regarding the time parameters, differences in the halftime of the aggregation process and its endpoint were observed. The control reaches the *t*_1/2_ point after 17.17 h of incubation. In contrast, the active compounds reach that point later, after 19.02 and 18.66 h, in the case of compounds 18 and 22. A similar time, 19.67 h, characterizes the halftime of the aggregation process in the presence of the reference inhibitor oleuropein aglycone. The trends regarding the aggregation endpoint are the same. It could be implied that the active compounds slow down the aggregation process.

### 3.3 Solvent Accessibility of the Peptide’s *C*-terminus during Amylin Aggregation and Binding Studies of Compounds 18 and 22

Utilizing the intrinsic fluorescence of aromatic amino acids such as tryptophan (Trp), tyrosine (Tyr), and phenylalanine (Phe) offers a powerful approach for site-specific analysis of protein folding and aggregation processes.^48,49^ Precisely, amylin contains three fluorescent residues that are critical for such studies: Phe15, Phe23, and Tyr37. Considering that tyrosine 37 emits fluorescence detectable at 278 nm, leveraging this emission can be utilized to explore the conformational alterations induced by amylin aggregation. Following incubation, the amylin peptides showed a peak in fluorescence intensity (Figure 2). Subsequently, the fluorescence intensity decreased in a nonlinear manner. The observed characteristics indicate that significant and rapid local changes occurred during amylin aggregation, particularly during the lag phase.^41,81^ In fact, when amylin is aggregating, the fluorescence is decreased due to the reduced exposure of the residues to the solvent because of the conformational changes presented with the stacking of the protein explained by the interactions between Tyr 37 and Phe 15 between different amylin monomers during nuclei/small aggregates formation, which give less access to the tyrosine at position 37.^41,44,82^ Moreover, Figure 2 demonstrates a reduction in fluorescence intensity in the presence of e active compounds (compounds 17, 18, 21, and 22). This decrease is more pronounced during the first hours of incubation, corresponding to the lag phase identified previously, which can explain the results obtained from kinetic parameters. It can be concluded that the change in fluorescence intensity observed during protein aggregation in the presence of various compounds reflects direct quenching due to protein conformational changes, mainly during the lag phase determined by ThT experiments. These changes result from the interaction of the amphipathic compounds with the hydrophilic and hydrophobic regions surrounding Tyr37 in amylin.

**Figure 2:**
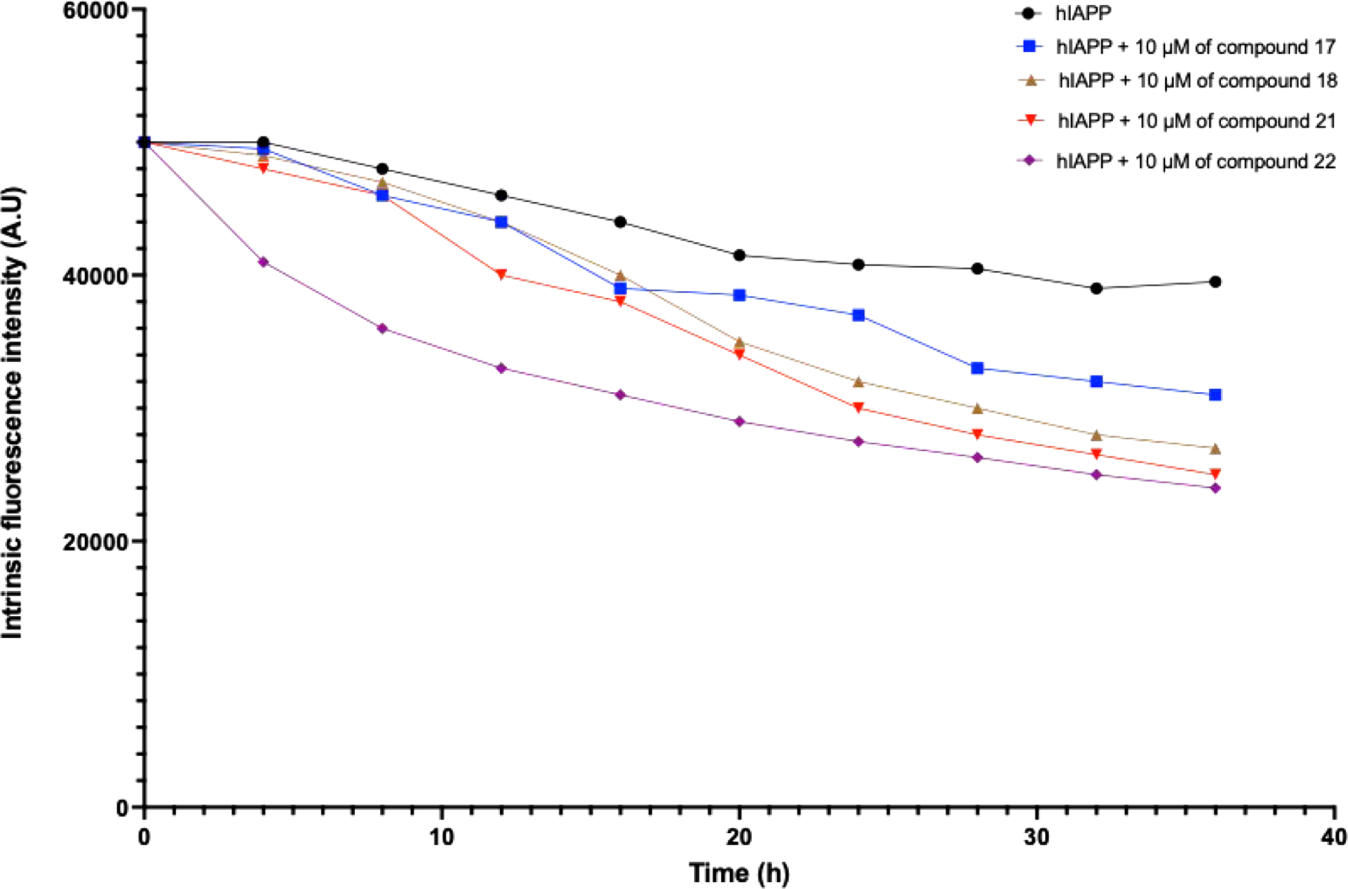
Temporal evolution of the intrinsic fluorescence intensity of amylin during the aggregation process in the presence or absence of 10 μM compounds 17, 18, 21, or 22. The values of the fluorescence quantum yield, calculated as the area under the emission spectra, are presented as means ± SEM of quadruplicate determinations obtained from three independent experiments. Sample fluorescence was determined by subtracting the fluorescence of the buffer.

To further investigate the involvement of Tyr37 in the interaction between amylin and the compounds, we measured the fluorescence of amylin excited at 275 nm in the presence of the most effective compounds, 18 (Figure 3A) or 22 (Figure 4A). The results of fluorescence quenching of amylin at 25 °C showed that the fluorescence intensity around peak 340 nm decreased as the concentrations of compounds 18 and 22, which were shown to be more efficient in inhibiting amylin aggregation, increased from 0 to 20 μM. However, there were no significant changes in the maximum emission wavelength or the shape of the peaks. This demonstrated that both compounds 18 and 22 caused a substantial quenching of amylin’s intrinsic fluorescence due to the interaction, suggesting induced conformational changes. Notably, no spectral shifts were observed in the emission spectra upon formation of the amylin–compound complex, suggesting that Tyr residues did not change polarity and there were no major conformational changes in the Tyr37 region of the amylin.

**Figure 3:**
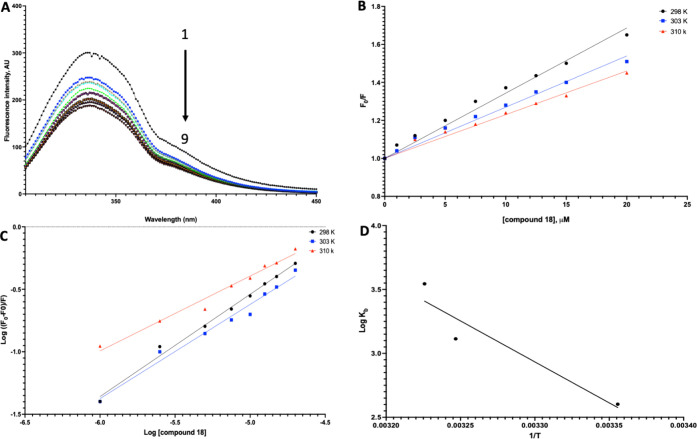
Binding studies of compound 18. A) Quenching spectra of amylin (15 μM) intrinsic fluorescence by increasing the amount of compound 18 at 25 °C. The arrow indicates the increase in compound 18 concentration (0–20 μM). B) Stern–Volmer plot for the quenching of amylin by compound 18 at 25/30/37 °C). C) Plot of log­[(*F*_0_ – *F*)/*F*] vs log [compound 18] for the quenching process of compound 18 with amylin at 25/30/37 °C, used to calculate the number of binding sites in the amylin–compound 18 system. D) Van’t Hoff plot of amylin–compound 18 system.

**Figure 4:**
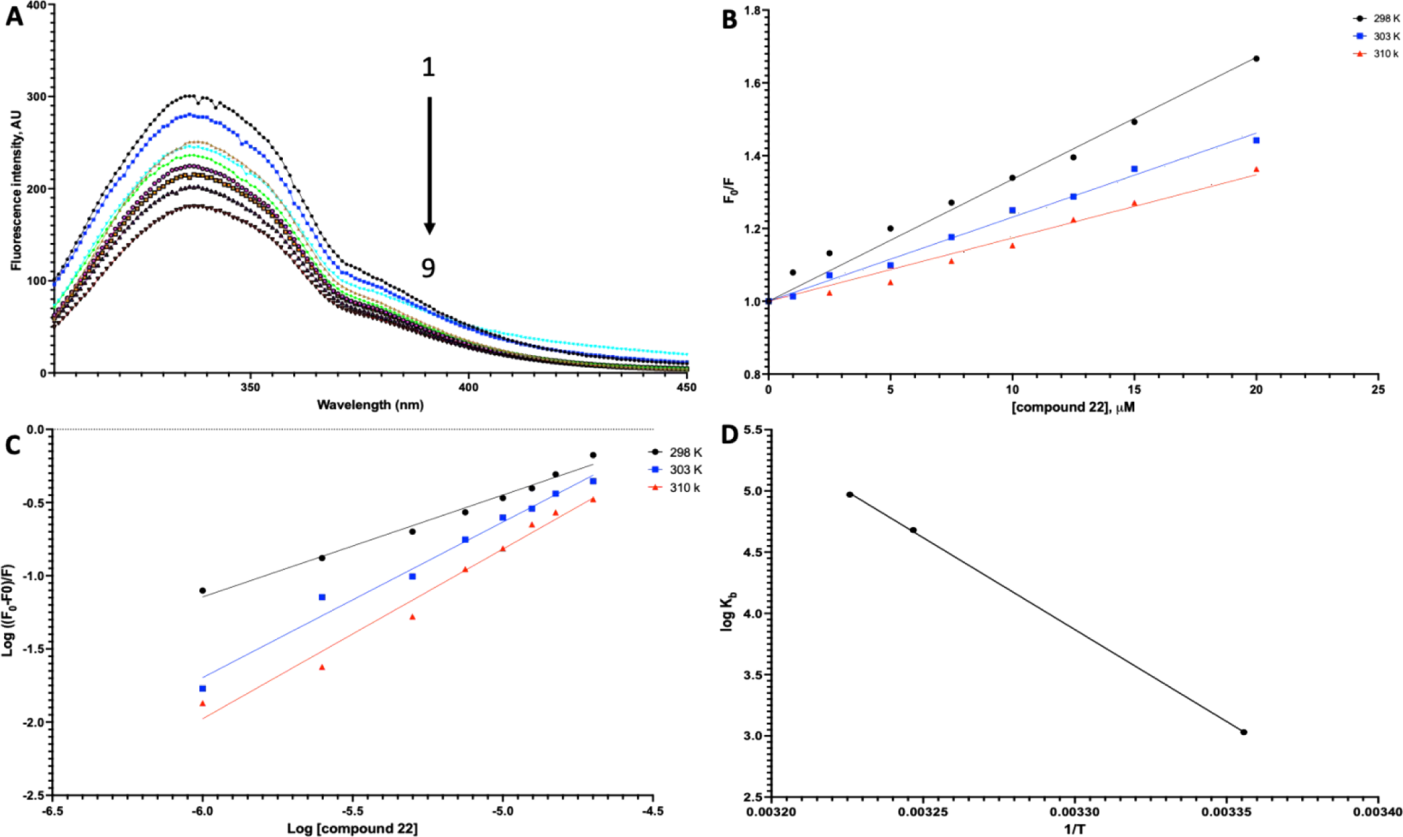
Binding studies of compound 22. A) Quenching spectra of amylin (15 μM) intrinsic fluorescence by increasing amounts of compound 22 at 25 °C. The arrow indicates the increase in compound 22 concentration (0–20 μM). B) Stern–Volmer plot for the quenching of amylin by compound 22 at 25/30/37 °C. C) Plot of log­[(*F*_0_ – *F*)/*F*] versus log­[compound 22] for the quenching process of compound 22 with amylin at 25/30/37 °C, used to calculate the number of binding sites in the amylin–compound 22 system. D) Van’t Hoff plot of amylin–compound 22 system.

Next, we applied the Stern–Volmer equation to interpret the quenching mechanism for protein–ligand interactions involving amylin and compounds 18 and 22. In parallel, we examined the temperature-dependent quenching constants (Table 4) and the collision quenching constant (*K*_q_) to elucidate the quenching mechanism. We observed lower Stern–Volmer constant (*K*_sv_) values at higher temperatures, indicating the formation of a nonfluorescent and static complex. The values of *K*_q_ for the amylin complexes with the compounds were more significant than the maximum theoretical value for dynamic quenching processes (2 × 10^10^ M^–1^ s^–1^), further supporting the presence of static quenching in these interactions.

**Table 4:** Quenching Measurements at Different Temperatures

	Stern–Volmer quenching constants					
	10^4^ *K*_sv_ (M^–1^)		10^12^ *K*_q_(M^–1^ s^–1^)		*R* ^2^	
Temperature (°C)	Compound 18	Compound 22	Compound 18	Compound 22	Compound 18	Compound 22
25	3.43 ± 0.21	3.35 ± 0.18	5.80 ± 0.17	5.67 ± 0.16	0.9921	0.9926
30	2.70 ± 0.39	2.30 ± 0.27	4.57 ± 0.11	3.89 ± 0.11	0.9923	0.9912
37	2.30 ± 0.18	1.70 ± 0.13	3.90 ± 0.12	2.88 ± 0.12	0.9917	0.9879

Finally, we determined the binding constant (*K*_b_) to evaluate how temperature affects the strength and longevity of the interaction between amylin and compounds 18 and 22, with a higher *K*_b_ indicating a stronger affinity. To achieve this, we calculated the binding constant (*K*_b_) between amylin and the compounds, where a higher *K*_b_ correlates with a stronger interaction. We found that both compounds strongly interacted with amylin (*K*_b_ = 10^4^ M^-1^). Furthermore, *K*_b_ increased with temperature, suggesting that the stability of the interactions improved as the temperature rose. In comparing the interactions of amylin with compounds 18 and 22, we observed that compound 22 exhibited higher *K*_b_ and lower IC_50_ values than compound 18. For the number of quenching sites, observed *n* values (0.6–1.15) reflect sample heterogeneity and model deviations typical for amyloid systems and should not be interpreted as stoichiometric site counts. Nevertheless, we noted that the value of *n* is greatest for compound 22 (Table 5) which again points to its specific binding properties, mainly at temperatures of 30 and 37 °C. These results suggest that compound 22 interacts more strongly with amylin than compound 18, mainly at physiological temperature (37 °C).

**Table 5:** Binding Parameters at Different Temperatures

	Binding parameters					
	*n*		10^4^ *K*_b_ (M^–1^)		*R* ^2^	
Temperature (°C)	Compound 18	Compound 22	Compound 18	Compound 22	Compound 18	Compound 22
25	0.60 ± 0.12	0.80 ± 0.15	0.04 ± 0.21	0.10 ± 0.39	0.9878	0.9850
30	0.75 ± 0.10	1.06 ± 0.10	0.13 ± 0.18	4.78 ± 0.32	0.9916	0.9812
37	0.92 ± 0.10	1.15 ± 0.15	0.35 ± 0.22	9.33 ± 0.38	0.9796	0.9770

Elucidating the relationship between thermodynamic parameters Δ*H*, Δ*S*, and Δ*G* and binding is imperative in investigating the spontaneity and strength of the interactions between the amylin and compounds 18 and 22. Usually, four noncovalent forces are involved in the protein–ligand interaction: hydrogen bonding, hydrophobic interactions, van der Waals, and electrostatic interactions.^83^ In Table 6, Δ*H* > 0 and Δ*S* > 0 suggest a significant influence of hydrophobic forces in the interaction between amylin and both compounds. Negative Δ*G* indicates a spontaneous binding interaction^84^ for both complexes, which increases with the increase in temperature (Figures 3D, 4D). The Δ*G* values for compound 22 are more negative than those for compound 18, suggesting that the bonding between compound 22 and amylin involves more hydrophobic interactions and is more spontaneous than for the complex amylin–compound 18.

**Table 6:** Thermodynamic Parameters of Compound Interactions with Amylin at Different Temperatures

	Thermodynamic parameters					
	Δ*G* (kJ/mol)		Δ*H* (kJ/mol)		Δ*S* (J/mol K)	
Temperature (°C)	Compound 18	Compound 22	Compound 18	Compound 22	Compound 18	Compound 22
25	–5.99 ± 0.10	–5.05 ± 0.27	53.31 ± 4.90	124.00 ± 4.50	199.00 ±14.13	433.00 ± 9.13
30	–6.99 ± 0.20	–7.19 ± 0.17				
37	–8.38 ± 0.30	–10.23 ± 0.41				

The investigations into the interactions between amylin and the most active inhibitors (compounds 18 and 22) revealed key insights into their binding and thermodynamic properties. Experiments revealed that these two compounds induced significant quenching of amylin’s intrinsic fluorescence without affecting the emission spectra, suggesting conformational changes without major alterations in the Tyr37 region. We observed lower Stern–Volmer constant (*K*_sv_) values at higher temperatures, indicating the formation of a nonfluorescent and static complex. When examining binding constants, we observed that compound 22 exhibited a higher *K*_b_ with higher *n* and lower IC_50_ values compared to compound 18. Further analysis of the thermodynamic properties of these interactions revealed positive enthalpy and entropy values, suggesting a significant influence of hydrophobic forces in the interaction between amylin and both compounds, with compound 22 binding involving more hydrophobic interactions.

### 3.4 Cell Viability

Given the association between amylin aggregation and pancreatic cytotoxicity,^44^ we tested the cytotoxic effects of amylin aggregates on pancreatic cells and assessed whether specific compounds could inhibit this toxicity. The first experiment used amylin aggregates, removed at different incubation times, to incubate with INS-1E cells. An MTT test was performed, which showed that the degree of cytotoxicity depends on the size of the introduced aggregates. As seen in Figure 5A, the most significant loss of cell viability was present within small and medium aggregates compared to monomeric amylin and control samples. This test indicated that amylin is more toxic during the initial lag phase of aggregation than in the exponential phase, suggesting that amylin tends to exhibit heightened toxicity during the initial stages of aggregation.^85^ Next, INS-1E cells were cultured for 24 h in the presence of amylin aggregates and aged for 36 h in the absence or presence of 1 μM oleuropein aglycone (used as a control), 3.46 μM of compound 17, 2.92 μM of compound 18, 3.31 μM of compound 21, and 2.61 μM of compound 22. At the end of the incubation, we performed a cytotoxicity assay. We found that cells incubated with compound 22 showed the highest viability, nearly 93% (around 8% points more than the oleuropein aglycone). Compounds 18 and 21 also prevented cytotoxicity comparably to the oleuropein aglycone, with cell viability approximately 30% points higher than the sample with amylin fibrils (Figure 5B).

**Figure 5:**
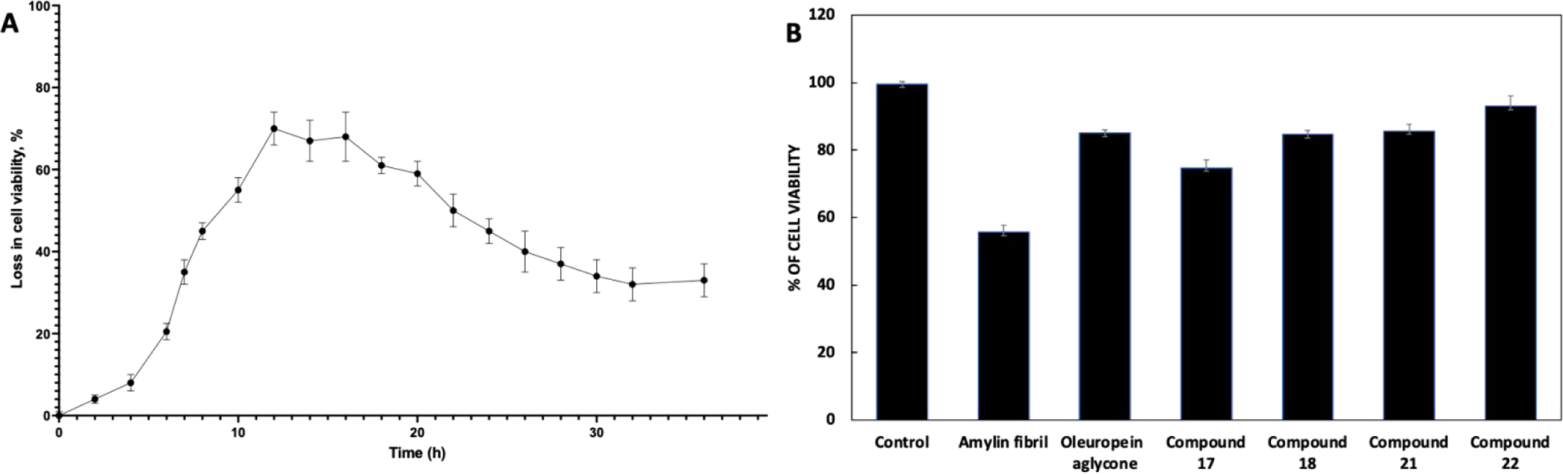
Selected aggregation inhibitors’ effect on amylin cytotoxicity. A) Kinetics of viability of INS-1E cells upon exposure to amylin aggregates. The final protein concentration within the culture medium was 5 μM. The cells were incubated for 24 h. Cell viability is expressed as the percentage of 3-(4,5-dimethylthiazol-2-yl)-2,5-diphenyltetrazolium bromide (MTT) reduction using cells treated with the same volume of buffer as a reference (100% 3-(4,5-dimethylthiazol-2-yl)-2,5-diphenyltetrazolium bromide reduction). The values (averages ± S.D.) are obtained from three independent experiments.

The cytotoxicity assays show that small and medium aggregates cause the most significant loss of cell viability, indicating the heightened toxicity of amylin in the initial lag phase of aggregation. When INS-1E cells were cultured with the MTDLs, the results showed that compounds 18, 21, and 22 retained cell viability, with compound 22 being the most effective at preventing the cytotoxic effects of amylin on pancreatic cells.

B) Amylin cell viability in the presence and absence of the IC50 concentration of the different selected inhibitors was compared with oleuropein aglycone (positive control). The cells were cultured for 24 h in the presence of amylin aggregates and aged for 36 h in the absence or presence of oleuropein aglycone and compounds 17, 18, 21, and 22, diluted 1:20 in the culture medium. At the end of the incubation, an MTT assay was performed.

### 3.5 *In Silico* Study

The *in silico* study aided in understanding the amylin aggregation process and aggregation inhibition. It comprised building amylin molecular systems, molecular docking, and molecular dynamics simulations of amylin systems solely and with compounds. We selected two *in vitro*-tested compounds for detailed analysis: the most active compound based on ThT assay (compound 18 with 91.5% of aggregation inhibition) and the least active one (compound 9 with 4.3% of aggregation inhibition). We designed our amylin molecular systems to represent different aggregation phases. Therefore, we built models of initiatory monomer associations and mature amyloid fibrils. For a single amylin monomer, we selected a peptide NMR structure (PDB ID: 2L86) reflecting the characteristics of the amylin used in the *in vitro* studies (37 amino acids, an amidated *C*-terminus, and a disulfide bridge between residues 2 and 7). Using protein–protein docking, we built associations of up to five amylin helical peptides from this starting structure. For amyloid fibril models, we chose five amylin β-strands (PDB ID: 7M65) and combined this model with one additional helical amylin monomer. We sought to establish how the amylin systems behave throughout the simulation as our baseline and compare them to the respective simulations with the two selected compounds. We were particularly interested in the structural changes of amylin in various molecular systems and whether the compounds could influence them.

We started by docking all MTDLs to the amylin systems to see the overall trends. The docking site was consistent across all of the compounds. To analyze the results further, we focused on two selected compounds. In the docking to the first amylin system, which involved one helical monomer, compounds 18 and 9 exhibited lower binding energy in the *S* absolute configuration; therefore, this stereoisomer was chosen for the analysis in the subsequent systems for consistency.

Next, we studied the monomeric amylin structure system in detail. The peptide exhibits two α-helical fragments: one encompassing residues Cys7–Val17 and the other from Asn21 to Ser28. Molecular docking studies revealed that compounds interact with amino acids in these regions. Compound 18 interacts with Asn22 through a hydrogen bond and Phe15 through π–π stacking (Figure 6A), whereas compound 9 interacts with Asn22 only through hydrogen bonds (Figure 6B), lacking a π–π interaction with any aromatic amino acid of amylin. Molecular docking results suggest that the active compounds may be able to create more interactions with amylin that exploit the peptide’s hydrophobicity compared to the inactive compounds.

**Figure 6:**
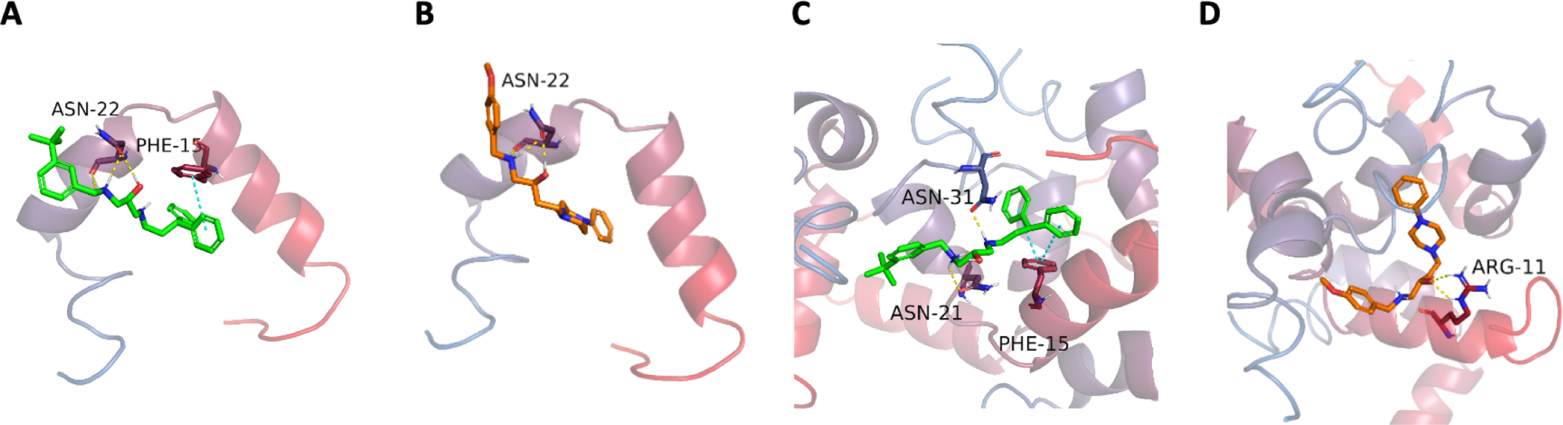
Molecular docking results: A) Compound 18 bound to the one-monomer amylin system. B) Compound 9 bound to the one-monomer amylin system. C) Compound 18 bound to the five-monomer amylin system. D) Compound 9 bound to the five-monomer amylin system.

Amylin monomer solely and two complexes with compounds 18 and 9 were subjected to molecular dynamics simulation to observe the system’s evolution over time. To describe the results comprehensively, we visualized the systems at the beginning and end of the simulation. Moreover, we calculated the secondary structure timeline diagrams and amino acid conformations. Visual representation of the systems at the start and end of the simulation (Figure 7) shows a similar tendency for the α-helical fragment relaxation in the middle part of the amylin monomer following the molecular dynamics simulation in two systems: the amylin monomer solely and with compound 9 (indicated with arrows in Figure 7). Conversely, compound 18 stabilizes the α-helical conformation of amylin in this region. It even introduces new helical fragments absent before the simulation (residues 17–19 and 30–37, indicated with arrows in Figure 7), implying that active compounds can maintain and facilitate α-helix formation in amylin peptides. Furthermore, when comparing the visual representations of systems with compounds 18 and 9, it is clear that only compound 18 remained attached to the amylin monomer, while compound 9 partially relocated from the peptide, which suggests that active compounds are more likely to stay attached to the amylin monomer compared to their inactive counterparts.

**Figure 7:**
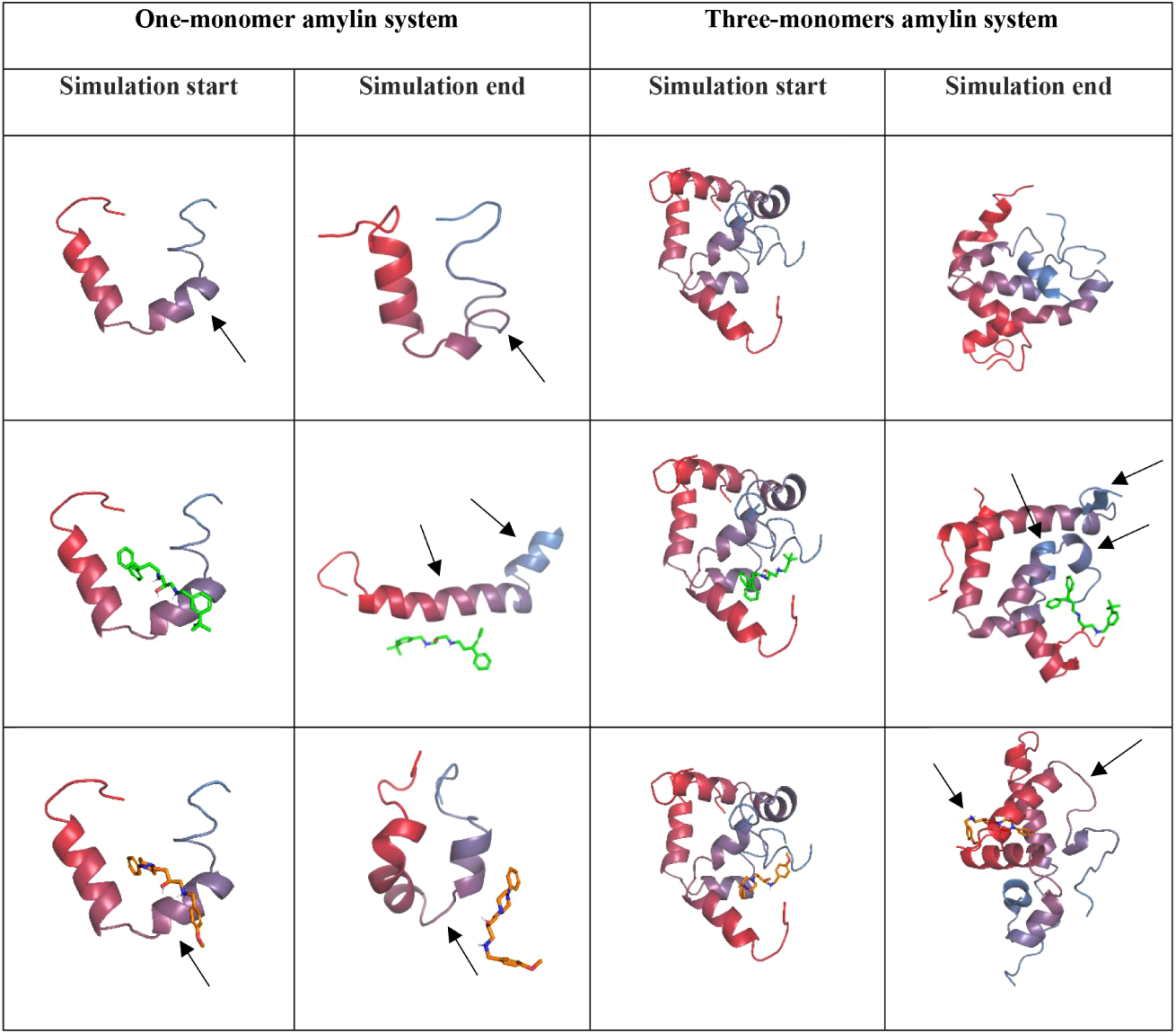
Visualizations of amylin structures at the start and the end of molecular dynamics simulations: compound 18 in green, compound 9 in orange, *C*-terminus in blue, *N*-terminus in red.

The secondary structure timeline diagrams (Figure 8) revealed detailed changes in the peptide’s secondary structure. For the amylin system solely, the chart shows that the initial α-helix in the middle part of the amylin monomer transitions into turns and isolated bridges, which serve as intermediate structures for future β-sheet formation (Figure 8A). Notably, this structural alteration expands over a longer fragment of the amylin monomer, residues 17–37, corroborating existing literature that suggests the involvement of the 20–29 fragment in the early aggregation process.^32,33^ Furthermore, we noticed similarities between the amylin system solely and the system with compound 9 regarding residues 19–21 (yellow rectangles in Figure 8) and 29–37 (brown rectangles in Figure 8) conformations, which are mainly 3_10_-helices and turns. This contrasts with the system containing compound 18, where the secondary structure timeline diagram shows that these amylin amino acids adopt the α-helix conformation (compare Figure 8B with Figures 8A and C). These tendencies are further elucidated in the three systems’ amino acid conformation count (Figure 9). Only the system with compound 18 exhibited an increase in amino acids in the α-helix conformation compared to the initial structure, maintaining a level of 25–30 throughout the entire simulation (Figure 9B). In the systems of amylin solely and with compound 9, the count of amino acids in the α-helix conformation decreased to less than 10 by the end of the simulation, followed by an increase in the isolated bridge conformations (Figure 9A and C). This observation could indicate that systems with the amylin peptide solely or combined with inactive compounds are more likely to develop amino acid conformation changes, which contribute to the formation of future amyloidogenic structures.

**Figure 8:**
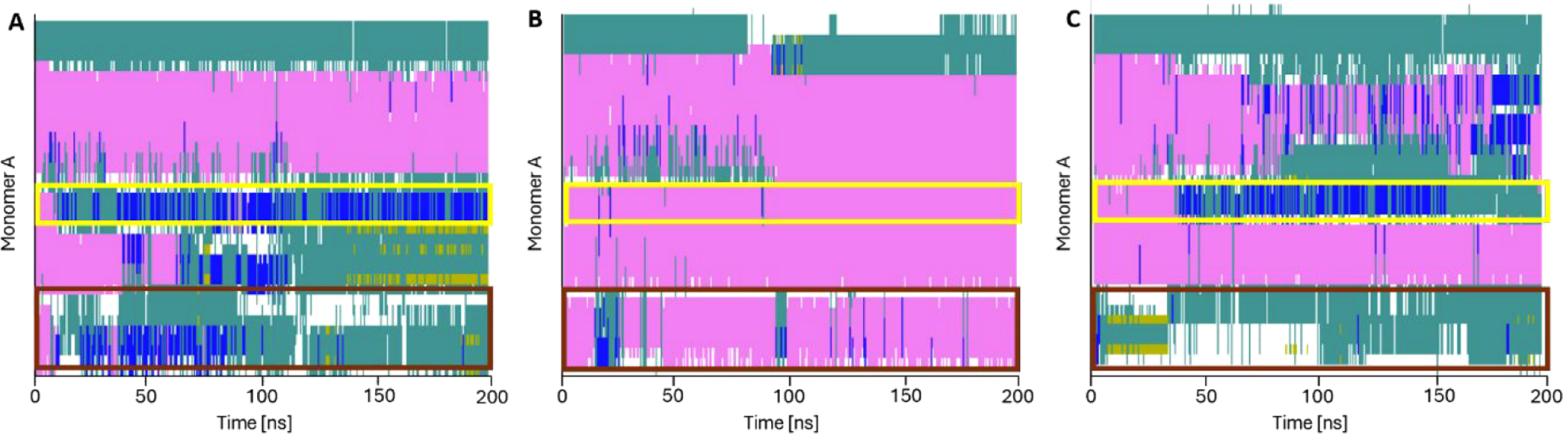
Secondary structure analysis through the 200 ns molecular dynamics simulation of the one-monomer amylin system. A) Control (amylin), B) amylin with compound 18, C) amylin with compound 9; α-helix in pink; 3_10_-helix in blue; turn in dark green; isolated bridge in light green, coil in white.

**Figure 9:**
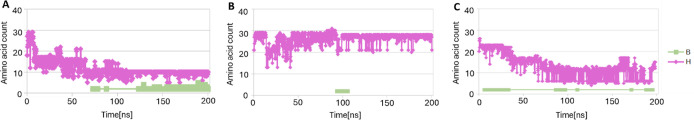
Changes in the number of amino acids in the α-helical (H) and isolated bridge (B) conformations in the one-monomer amylin system: A) Control (amylin), B) amylin with compound 18, and C) amylin with compound 9.

Next, we addressed the dimer complex of amylin with regard to building the structure and performing molecular docking. The dimer complex of amylin is formed through hydrophobic interactions between the helical fragments of two monomers (chains A and B in Figure 10A). As indicated in the literature,^32,33^ aromatic amino acids, particularly Phe15 and Phe23, participate in π–π stacking, facilitating the association of amylin monomers (Figure 10A). In molecular docking to the amylin dimer, compound 9 formed fewer interactions with the peptides than compound 18. Compound 18 interacted with three residues, in contrast to only one interaction formed by compound 9, repeating the previous tendency observed in the one-monomer amylin system.

**Figure 10:**
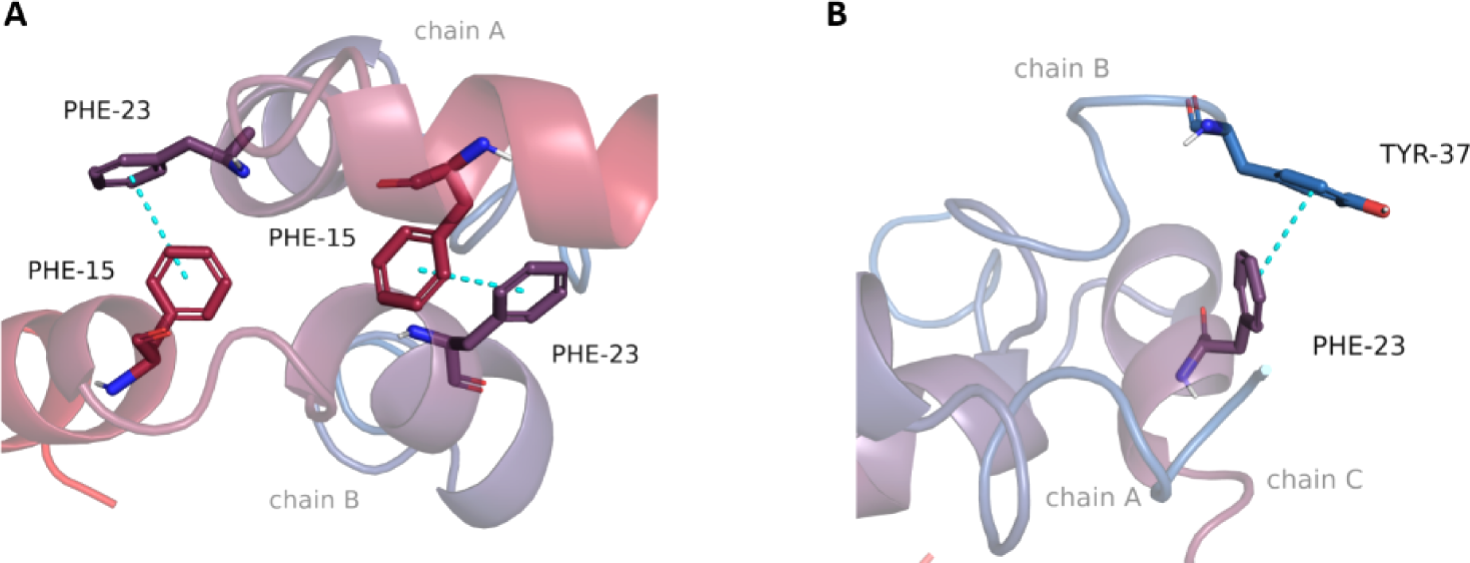
Hydrophobic interactions between amylin monomers: A) The two-monomer system of amylin and B) the three-monomer system of amylin.

Subsequently, we examined the structure of the amylin trimer. The structure incorporates another amylin monomer, facilitating π–π stacking interaction between two aromatic amino acids, Phe23 and Tyr37 (Figure 10B). The association is further stabilized by hydrogen bonds among other amino acids. In molecular docking, compounds 18 and 9 create the same number of interactions with the amylin trimer. Both compounds form hydrogen bonds with Asn31. Additionally, compound 18 creates an π–π interaction with Phe15, whereas compound 9 forms a π–cation interaction with Arg11. We were particularly interested in molecular dynamics simulations of higher amylin associations regarding the aggregation process. Since the 200 ns simulations of the three-monomer amylin systems revealed interesting and distinctive observations, we decided to simulate them for a longer time (500 ns) to further observe the systems’ evolution.

The results of our simulations provided valuable insight into the behavior of a higher association of amylin peptides. A visual representation of the systems at the start and end of the simulation (Figure 7) shows the differences in amino acid conformations at the *C*-termini of the amylin monomers. In the systems of amylin solely and with compound 9, the *C*-termini amino acids occur in the turn or coil conformations both at the start and at the end of the simulation. Contrastingly, in the system with compound 18, α-helical fragments appear at the *C*-termini at the end of the simulation (indicated with arrows in Figure 7). Additionally, we noticed that in the system with compound 9, there was a change in the middle part of one of the amylin monomers (indicated by an arrow in Figure 7), where the α-helix in the starting structure transitioned into the coil, which did not happen in any other system. This may suggest that the inactive compounds facilitate noticeable α-helix disruption, a tendency previously seen in the systems of one amylin monomer. Finally, we noticed the relocation of compound 9 from its initial position to the *N*-termini of amylin (indicated by an arrow in Figure 7). Once more, this is a similar observation to the one we made regarding the systems with one amylin monomer, where compound 18 remained bound to amylin around the initial position and compound 9 changed its location.

The secondary structure timeline diagrams (Figure 11) highlight the structural differences between the three systems. Our analysis focuses on residues 21–37 in monomer A (yellow rectangles in Figure 11) and residues 13–23 in monomer C (brown rectangles in Figure 11). Regarding the region highlighted in yellow, only the system with compound 18 maintains the α-helical conformation of most of the residues in this area. In the amylin system solely, only about half of the residues in the same region demonstrate α-helical conformations. Finally, in the system with compound 9, most residues transition into turn or coil conformations. We observed a similar tendency in the amylin region indicated in the brown rectangle, where the most α-helical amino acid conformations occur in the system with compound 18 (compare Figure 11B with Figure 11A and C). The α-helical conformation count presented in Figure 12 quantitatively describes these differences. The system with compound 18 represents a stable α-helical conformation count of around 70 amino acids (Figure 12B). For the two other systems, there was a significant drop in the α-helical conformation count at the beginning of the simulation to around 60 (solely amylin system) and 50 (system with compound 9) amino acids (Figure 12A and C). Regarding the isolated bridge conformation, the most viable increase is seen in the compound 9 system. These tendencies are consistent with the quantitative amino acid conformation count we performed for one-monomer amylin systems.

**Figure 11:**
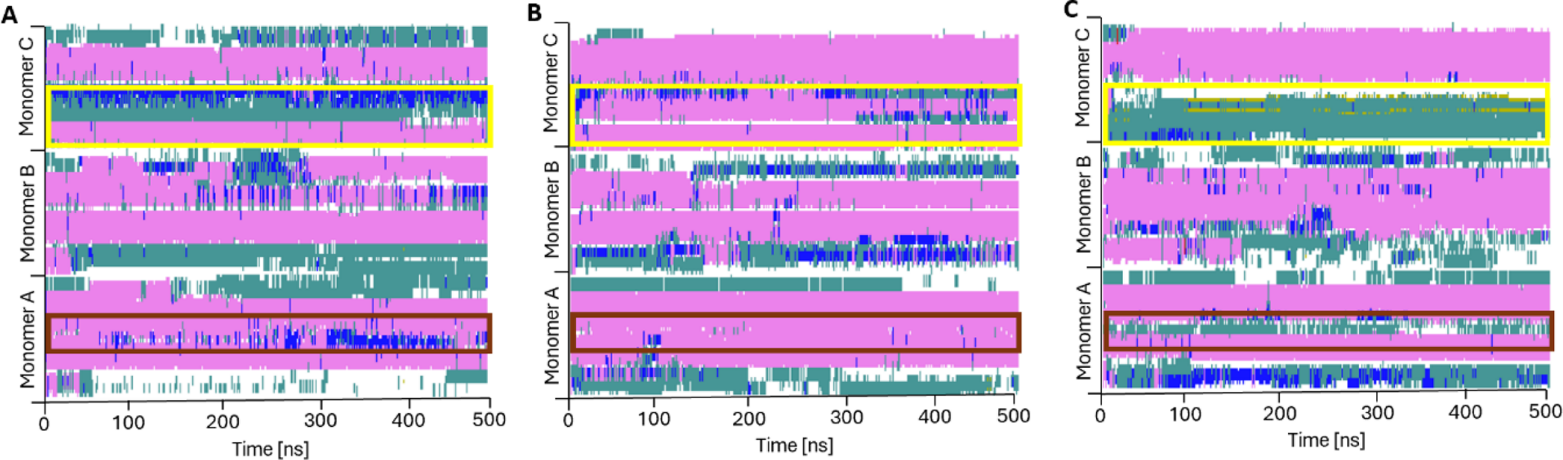
Secondary structure analysis through the 500 ns molecular dynamics simulation of the three-monomer amylin system: A) Control (amylin), B) amylin with compound 18, C) amylin with compound 9; α-helix in pink; 3_10_-helix in blue, turn in dark green, isolated bridge in light green, coil in white.

**Figure 12:**
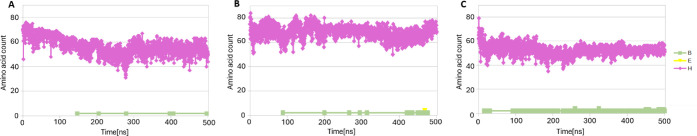
Changes in the number of amino acids in the α-helical (H), isolated bridge (B), and β-sheet (E) conformations in the three-monomer amylin system: A) Control (amylin), B) amylin with compound 18, and C) amylin with compound 9.

The pentameric amylin system is composed of five helical monomers of amylin. This system demonstrates more extensive interactions between the peptides, mainly multiple π–π contacts between aromatic amino acids. In molecular docking, this system showed a clear tendency for more amino acids to interact with compound 18 than with compound 9. Specifically, compound 18 interacted with Asn21 and Asn31 through hydrogen bonds and Phe15 through double π–π stacking (Figure 6C), while compound 9 interacted only with Arg11 (Figure 6D). Moreover, compound 18 was positioned deeper within amylin’s helical fragments than compound 9. These findings add to previous observations obtained through molecular docking, suggesting that the active compounds may be able to form more polar and hydrophobic interactions with the amylin monomers than their inactive counterparts.

Next, we addressed the models of amyloid fibrils, which comprised five amylin β-strands and five amylin β-strands, with the addition of one helical monomer. Since the secondary structure of amylin monomers seemed to be tendentiously influenced by compounds throughout the simulation timeline in previously discussed models (Figures 8 and 11), we wondered if compound 18 would have the ability to change the β-sheet conformation in already formed aggregates into the initial α-helix and stabilize the α-helical conformation of a monomer when added to mature amyloid fibrils. As with all systems, we started with molecular docking to examine the interactions of compounds with aggregates.

First, in molecular docking to the system comprising five amylin β-strands, we observed the tendency of more amino acids to form contacts with compound 18—two interactions with Ser29 and Thr36 compared to only one with Asn31 for compound 9. Both compounds were located in the *C*-terminal region of amylin, with compound 18 surrounded by substantially more hydrophobic residues than compound 9.

Second, in molecular docking to the amyloid fibril model comprising five β-strands and one α-helical form of the peptide, both compounds formed hydrogen bonds with one amino acid: compound 18 with Thr36 and compound 9 with Ser29. However, the differences in binding modes became apparent in the occupation of the hydrophobic pocket. Compound 18 spread evenly across the molecular surface, forming numerous hydrophobic contacts. Conversely, compound 9 occupied only half the available space in the hydrophobic pocket, lacking potential interactions.

Nevertheless, molecular dynamics simulations of these systems, both solely and with compounds, did not show consistent differences. Although we observed some occasional trends, such as compound 18 stabilizing the α-helical conformation of the amylin monomer for a longer time compared to compound 9 or inducing conformational changes in the amylin β-strand, the differences between the compounds in these systems were not as pronounced as those observed in previous amylin associations

Overall, the *in silico* study comprised building amylin molecular systems, molecular docking of the *in vitro*-tested compounds, and molecular dynamics simulations. The building of amylin molecular systems showed the importance of hydrophobic interactions, especially π–π stacking of amylin amino acids Phe15, Phe23, and Tyr37 as a driving force in monomer association. Molecular docking depicted the interactions of *in vitro*-tested compounds with amylin systems. Compound 18 (an *in vitro* active compound) tended to create more interactions with amylin peptides compared to compound 9 (an *in vitro* inactive compound). It fully exploited the hydrophobicity of amylin peptides, suggesting the importance of lipophilic properties in ligand design. Molecular dynamics simulations of amylin monomer associations, both solely and with compounds 18 and 9, brought interesting insights into the process of amylin aggregation and its possible inhibition. There were opposite conformational tendencies in the compound 18 systems vs systems with compound 9 and amylin solely. While systems with compound 18 tended to maintain the existing α-helical conformation and facilitated its formation, the systems of amylin solely or combined with compound 9 behaved oppositely, often causing α-helix disruption and transition into turns or isolated bridges, intermediate structures leading to future β-strands in amylin aggregates. Furthermore, compound 18 remained bound to the amylin structure around its initial position for a longer simulation time than compound 9, which tended to change its position and lose its interactions with the amylin structure. There were no distinctive tendencies during the simulation progression for the systems of mature amyloid fibrils. These systems likely need to be simulated for a substantially longer time scale.

## 4 Discussion

1-Benzylamino-2-hydroxyalkyl derivatives are multitarget-directed ligands (MTDLs) that have demonstrated effectiveness against Alzheimer’s disease (AD) targets.^42,43^ The compounds are categorized into two series: series A, in which the hydroxyalkyl amine nitrogen is incorporated into a piperazine ring, and series B, in which the same atom is part of an alkylamine fragment (Table 1). Both scaffolds underwent structure–activity relationship (SAR) analysis to identify the most effective combination of R_1_ and R_2_ substituents, as well as the number of carbon atoms linking the hydroxyl group to the nitrogen atom to maximize compound efficiency.

Series A favored aromatic substituents at the R_1_ position, particularly diphenylmethyl, combined with a bulky 3-*tert*-butyl group at R_2_. This combination produced compound 6, which exhibited over 50% inhibition of amylin aggregation in the ThT assay. Activity was further enhanced by extending the linker between the hydroxyl group and the nitrogen atom, resulting in compound 13, which is the most potent in Series A, with nearly 75% inhibition of amylin aggregation. This outcome aligns closely with the findings of Panek et al. in the ThT Aβ aggregation assay, where compound 13 demonstrated nearly 82% inhibition, making it the most active compound in that series.^42^

The insights gained from series A were applied to series B by introducing aromatic R_1_ substituents, such as 2,2-diphenylethyl and 3,3-diphenylpropyl, while maintaining the favorable 3-*tert*-butyl group at R_2_. As a result, four compounds from series B—17, 18, 21, and 22—exhibited over 88% inhibition of amylin aggregation in the ThT assay. Similarly to series A, this finding aligns with the results of the ThT Aβ aggregation assay,^42^ where the same compounds achieved over 84% inhibition of aggregation. The similarities between these studies suggest that 1-benzylamino-2-hydroxyalkyl derivatives are effective against both aggregating peptides Aβ and amylin. Among compounds 17, 18, 21, and 22, compound 22 demonstrated the lowest IC_50_ value, at 2.71 μM. However, in the studies conducted by Panek et al., the IC_50_ of compound 22 was approximately 1.5 μM lower, indicating a stronger inhibitory effect against Aβ aggregation.^42^

Evaluating the results further, when compounds 17, 18, 21, and 22 were incubated with amylin, DLS revealed that the average particle size of amylin remained approximately 10 nm at the end of the incubation period. This finding suggests that the most active compounds effectively prevent the usual increase in particle size linked to amyloid fibril formation. The cytotoxicity assays showed that the highest cell viability for the sample was obtained in the presence of compound 22 and was nearly 40% higher than that of the control. Furthermore, the active compounds’ mechanism of action was studied using the ThT assay, and kinetic parameters were calculated using the two-step autocatalytic aggregation model. The most significant differences between the control and active aggregation inhibitors were observed in the fibrillation constant and aggregation halftime. This result implied that the active compounds inhibited amylin aggregation mainly at the step of fibril elongation and slowed down the aggregation process. Due to significant quenching, the intrinsic fluorescence intensity of the amino acid tyrosine decreased mainly during the lag phase in the presence of the most active inhibitory compounds (18 and 22), indicating conformational changes without major modifications in the Tyr37 region. Stern–Volmer quenching constants (*K*_sv_, *K*_q_) confirmed the static quenching mechanism in the presence of compounds 18 and 22. Binding parameters indicated a strong interaction between amylin and both compounds, with a slightly more favorable interaction for compound 22. Because amylin can exist as a mixture of conformational states with varying accessibility of Tyr37, fitting to a single-class, independent-site model yielded fractional *n* values. The stability of the complexes increased as the temperature rose, particularly at 37 °C, suggesting that the compounds are efficient against aggregation under physiological conditions. Noncovalent hydrophobic forces involved in peptide–ligand interactions were shown to be crucial for binding. Furthermore, the binding process became more spontaneous with increasing temperature.^39,40^

Overall, the experimental methods employed in this study provided a comprehensive view of the amylin aggregation process. To investigate the early stages of aggregation at the level of monomers and small oligomers, we used fluorescence quenching assays to examine how aggregation inhibitors interact locally with specific peptide residues. This approach parallels mass spectrometry techniques commonly used to detect noncovalent peptide–inhibitor adducts.^86^ To probe later stages of aggregation, we employed DLS to assess changes in particle size and distribution indicative of oligomer and fibril formation. These measurements complement structural data obtained from circular dichroism, a widely used method for monitoring secondary structure transitions, such as α-helix to β-sheet conversion, during peptide aggregation.^86,87^ In addition, ThT fluorescence provided insights into the kinetics of fibril formation and inhibitor binding, while the MTT cytotoxicity assay assessed the functional consequences of aggregation on cell viability.

However, the *in vitro* nature of our assays limits our ability to infer the molecular mechanisms by which the compounds affect aggregation. In the absence of direct structural characterization techniques (such as cryo-EM and NMR), we performed *in silico* analyses to approximate peptide–inhibitor interactions. This enabled us to gain preliminary insight into the potential mechanism of action and to identify molecular features that distinguish active compounds from inactive ones. Our construction of amylin molecular systems highlighted the critical role of hydrophobic interactions between amylin helical fragments, particularly the amino acids Phe15, Phe23, and Tyr37, which drew monomers’ association.

Molecular docking provided further insight into how the most active and least active compounds bind to different amylin systems. This approach helped us observe specific binding tendencies that could explain the different levels of *in vitro* activity exhibited by the compounds we studied. Our observations indicate that compound 18 formed more interactions with amylin in most systems compared to compound 9. Regarding molecular interactions exhibited by compound 18, we found that van der Waals forces were the most prevalent, especially π–π interactions. Compound 9 rarely formed π–π interactions with amylin’s aromatic amino acids. Instead, it frequently created one or two hydrogen bonds with the peptide. Due to the bulky aromatic substituents of compound 18, it exploited the hydrophobic surface of amylin peptides more comprehensively than compound 9. Molecular dynamics simulation explored the dynamic behavior of amylin systems solely and with compounds. One of the most significant changes in amylin systems solely was the relaxation of peptide’s *C*-terminus. This transformation often involved the amino acid shifting from the α-helical conformation to turns or isolated bridges, known as intermediate structures leading to β-sheet formation. Over the course of the simulations, amylin systems demonstrated a general pattern of a decrease in α-helical conformation and an increase in isolated bridge conformations.

The simulations of amylin systems with bound compounds yielded significant insights that help explain the varied *in vitro* activities. Compound 18 was bound to amylin for a longer simulation time and remained close to the initial binding site, contrasting with compound 9, which changed its location in the systems of one and three amylin monomers. Additionally, compound 18 mostly stabilized the existing α-helical conformation of amylin and even introduced more helical fragments. This feature was not observed for compound 9, which often promoted amylin’s *C*-terminus relaxation to the 3_10_-helix, the turn, and the isolated bridge, contributing to future amyloidogenic conformations. However, it remains unclear whether *in vitro* active compounds can stabilize the α-helical monomer in the initial conformation when added to mature amyloid fibrils or influence the β-sheet structure of amylin. It is important to note that molecular dynamics simulations for aggregation processes require a significantly longer time scale than simulations for other systems, such as ligand–receptor binding. In this study, the longest simulation time was 500 ns, which was sufficient to observe the initial changes in the system but not enough to draw definite conclusions about process development.

## 5 Conclusions

In this study, we evaluated the efficacy of 1-benzylamino-2-hydroxyalkyl derivatives in inhibiting amylin aggregation by using a comprehensive approach that combined both *in vitro* and *in silico* methods. Through detailed SAR analysis, we identified the optimal scaffolds and terminal substituents for maximizing activity. Four compounds (17, 18, 21, and 22) demonstrated over 88% inhibition of amylin aggregation. Among them, compound 22 emerged as the most efficient, based on its IC_50_ value.

Additionally, compounds 16, 17, 18, 21, and 22 exhibited an average particle size of 10 nm according to the DLS method, suggesting their potential to efficiently interfere with the aggregation process. Further investigation into their mechanism of action revealed that the active compounds likely inhibit amylin aggregation at the fibrillation stage and delay the aggregation process.

Notably, compounds 18 and 22 also significantly quenched the intrinsic fluorescence of amylin without altering its emission spectrum, suggesting conformational changes that do not affect the Tyr 37 region. Binding and thermodynamic calculations confirmed strong interactions between amylin and these compounds, highlighting the critical role of hydrophobic interactions in their inhibitory activity. Additionally, compounds 18, 21, and 22 preserved the viability of INS-1E cells, with compound 22 proving to be the most effective at preventing the cytotoxic effects of amylin on pancreatic cells.

The molecular docking study showed that compound 18 formed more interactions with amylin peptides compared with compound 9. In molecular dynamics simulations, compound 18 consistently maintained and stabilized the peptide’s α-helical conformation, while compound 9 had the opposite effect, frequently disrupting the α-helix and promoting transitions into turns or isolated bridges. Moreover, compound 18 remained bound to the amylin structure near its initial docking position for a longer period, whereas compound 9 tended to shift position and lose its interactions with the amylin structure over time.

Altogether, the obtained results allowed us to pinpoint effective inhibitors of amylin aggregation, offering potential therapeutic avenues for conditions characterized by amyloid fibril formation and opening the door for potential MTDLs.

## Supplementary Material



## Data Availability

All data are included in this article.

## Notes

Includes p*K*_a_ values of ionizable groups; concentration–response curves for compounds 18 and 22 based on ThT assay (PDF)
