# Supplementary material for: Inhibition of Human Amylin Aggregation: In Silico and In Vitro Studies
Source: ACS Omega. 2025 Oct 31;10(44):52269–88. doi: 10.1021/acsomega.5c02443 (PMC12612973; doi:10.1021/acsomega.5c02443)
Supplement: Supplementary file 1 [file ao5c02443_si_001.pdf]

Supporting Information

Inhibition of human amylin aggregation: *in silico* and *in vitro* studies

Katarzyna Mizgalska[1,#], Ubaida Al.-Aani[2,#], Yaqoub Aljaidah[2,#], Dawid Panek[1], Ali Chaari[2,*], Marek Bajda[1,*]

[1] Department of Physicochemical Drug Analysis, Faculty of Pharmacy, Jagiellonian University Medical College, 30-688 Kraków, Medyczna 9, Poland

[2] Weill Cornell Medicine Qatar, Qatar Foundation, Education City, P.O. Box 24144, Doha, Qatar

[*] Corresponding authors: alc2033@qatar-med.cornell.edu, marek.bajda@uj.edu.pl

[#] these authors contributed equally on the manuscript

1. pKa values of ionizable groups

Table S1. pKa values calculated for the tested compounds using Epik from Schrodinger Suite

Compound	General structure	Substituents (R_1)	Substituents (R_2)	pKa_1	pKa_2	pKa_3
1	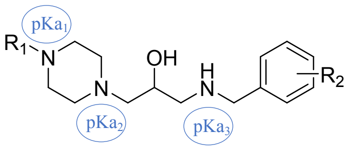 Compounds 1-7	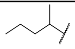 Pentan-2-yl	4-OCH_3	4.38	9.13	8.17
2		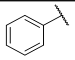 Phenyl	4-OCH_3	3.3	8.61	8.19
3		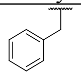 Benzyl	4-OCH_3	5.8	8.88	8.17
4		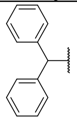 Diphenylmethyl	H	1.56	8.8	8.12
5		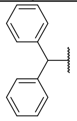 Diphenylmethyl	4-OCH_3	1.56	8.8	8.17
6		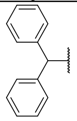 Diphenylmethyl	3-tert-butyl	1.56	8.8	8.14
7		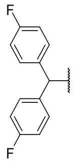 Bis(4-fluorophenyl)methyl	4-OCH_3	1.56	8.8	8.17

8	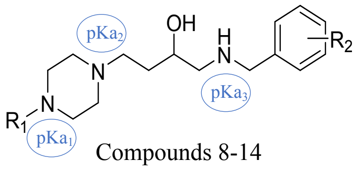<br>Compounds 8-14	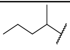<br>Pentan-2-yl	$4\text{-}OCH_3$	8.19	3.96	8.69
9		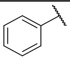<br>Phenyl	$4\text{-}OCH_3$	4.76	7.05	8.73
10		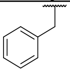<br>Benzyl	$4\text{-}OCH_3$	7.67	3.85	8.69
11		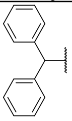<br>Diphenylmethyl	H	8.03	3.63	8.63
12		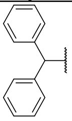<br>Diphenylmethyl	$4\text{-}OCH_3$	8.03	3.63	8.69
13		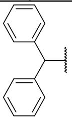<br>Diphenylmethyl	3-tert-butyl	8.03	3.63	8.65
14		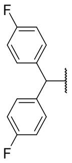<br>Bis(4-fluorophenyl)methyl	$4\text{-}OCH_3$	8.03	3.63	8.69
15	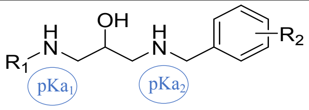<br>Compounds 15-18	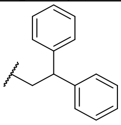<br>2,2-diphenylethyl	H	5.66	8.21	
16		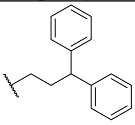<br>3,3-diphenylpropyl	H	6.04	8.22	
17		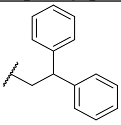<br>2,2-diphenylethyl	3-tert-butyl	5.66	8.23	
18		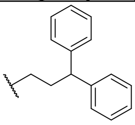<br>3,3-diphenylpropyl	3-tert-butyl	6.04	8.24	
19	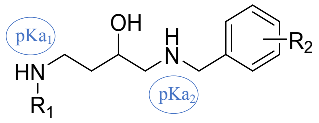<br>Compounds 19-22	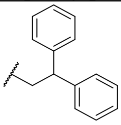<br>2,2-diphenylethyl	H	8.51	8.35	
20		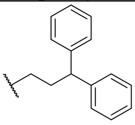<br>3,3-diphenylpropyl	H	8.88	8.35	

21		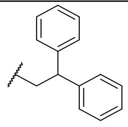 2,2-diphenylethyl	3-tert-butyl	9.46	8.55	
22		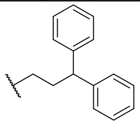 3,3-diphenylpropyl	3-tert-butyl	9.77	8.55	

2. Concentration-response curves for compounds 18 and 22 based on ThT assay

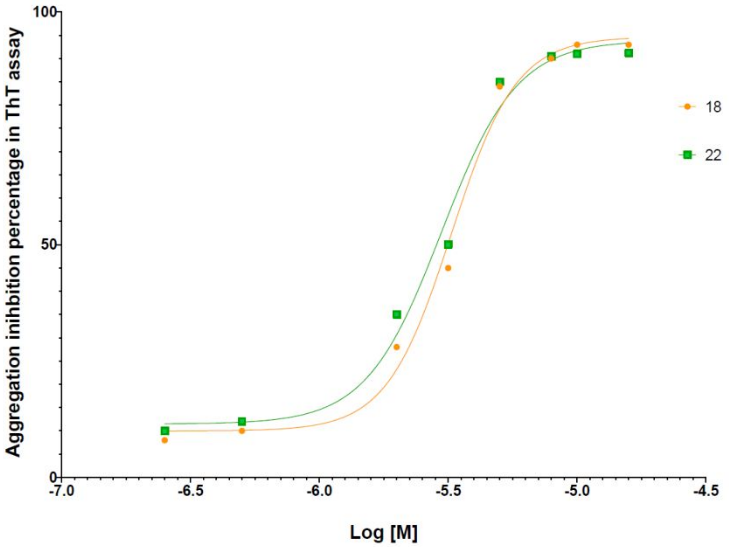

Figure S1. Concentration dependent inhibition curves used to determine the compounds' IC_{50}.
